# N6-methyladenosine-modified circSLCO1B3 promotes intrahepatic cholangiocarcinoma progression via regulating HOXC8 and PD-L1

**DOI:** 10.1186/s13046-024-03006-x

**Published:** 2024-04-20

**Authors:** Jing Li, Xiaohong Xu, Kaihao Xu, Xueliang Zhou, Kunpeng Wu, Yuan Yao, Zaoqu Liu, Chen Chen, Ling Wang, Zhenqiang Sun, Dechao Jiao, Xinwei Han

**Affiliations:** 1 Department of Interventional Radiology, The First Affiliated Hospital of Zhengzhou University, Zhengzhou, 450052 Henan China; 2 Interventional Institute of Zhengzhou University, Zhengzhou, 450052 Henan China; 3 Interventional Treatment and Clinical Research Center of Henan Province, Zhengzhou, 450052 Henan China; 4 Department of Colorectal Surgery, The First Affiliated Hospital of Zhengzhou University, Zhengzhou, 450052 Henan China

**Keywords:** Cholangiocarcinoma, circRNAs, Immune evasion, N6-methyladenosine, Tumor progression

## Abstract

**Background:**

Refractoriness to surgical resection and chemotherapy makes intrahepatic cholangiocarcinoma (ICC) a fatal cancer of the digestive system with high mortality and poor prognosis. Important function invests circRNAs with tremendous potential in biomarkers and therapeutic targets. Nevertheless, it is still unknown how circRNAs contribute to the evolution of ICC.

**Methods:**

CircRNAs in paired ICC and adjacent tissues were screened by circRNAs sequencing. To explore the impact of circRNAs on ICC development, experiments involving gain and loss of function were conducted. Various experimental techniques, including quantitative real-time PCR (qPCR), western blotting, RNA immunoprecipitation (RIP), luciferase reporter assays, RNA pull-down, chromatin immunoprecipitation (ChIP), ubiquitination assays and so on were employed to identify the molecular regulatory role of circRNAs.

**Results:**

Herein, we reported a new circRNA, which originates from exon 9 to exon 15 of the SLCO1B3 gene (named circSLCO1B3), orchestrated ICC progression by promoting tumor proliferation, metastasis and immune evasion. We found that the circSLCO1B3 gene was highly overexpressed in ICC tissues and related to lymphatic metastasis, tumor sizes, and tumor differentiation. Mechanically, circSLCO1B3 not only promoted ICC proliferation and metastasis via miR-502-5p/HOXC8/SMAD3 axis, but also eradicated anti-tumor immunity via suppressing ubiquitin-proteasome-dependent degradation of PD-L1 by E3 ubiquitin ligase SPOP. We further found that methyltransferase like 3 (METTL3) mediated the m6A methylation of circSLCO1B3 and stabilizes its expression. Our findings indicate that circSLCO1B3 is a potential prognostic marker and therapeutic target in ICC patients.

**Conclusions:**

Taken together, m6A-modified circSLCO1B3 was correlated with poor prognosis in ICC and promoted ICC progression not only by enhancing proliferation and metastasis via potentiating HOXC8 expression, but also by inducing immune evasion via antagonizing PD-L1 degradation. These results suggest that circSLCO1B3 is a potential prognostic marker and therapeutic target for ICC.

**Supplementary Information:**

The online version contains supplementary material available at 10.1186/s13046-024-03006-x.

## Introduction

ICC were heterogeneous malignancies that are known for being difficult to diagnose and treat [1]. Several Southeast Asian countries, particularly China and Vietnam, have reported a sharp rise in the prevalence of ICC in recent years [2]. Surgery or liver transplantation are potentially curative therapeutic choices for patients with early-stage disease, but tumor recurrence rates after resection remain demoralizing. While standard of care chemotherapy has proven benefits in advanced ICC, the effectiveness of chemotherapy and durability of their response are still quite limited [3]. Additionally, even though comprehensive genomic profiling investigations have identified clinically valuable alterations in several genes, such as FGFR2, IDH1, BRAF, NTRK, RET, and ERBB2, the prognosis for ICC remains unsatisfactory [4, 5]. Therefore, innovative methods are urgently required to find new treatment targets and clinically significant indicators to eventually increase patient survival with ICC.

Circular RNAs, which are covalently closed single-stranded RNAs, have recently regained popularity as a prevalent form of RNA species thanks to the progress in high-throughput RNA sequencing technology. Its unique circular conformation, which lacks 5’ m7G caps or 3’ poly (A) tails, and important function invest circRNAs with tremendous potential in biomarkers and therapeutic targets [6]. Up to date, there have been amounts of published reports highlighting circRNAs in the etiology and development of ICC through orchestrating microRNAs or decoying proteins, even interfering with signaling pathways [7–9]. Notably, circ-CCAC1 promoted tumor cell proliferation in CCA tissues and the permeability of vascular endothelial cell by up-regulating Yin Yang 1 and SH3GL2 expression in bile-resident exosomal [10]. Additionally, the Hippo and Wnt/-catenin pathways were stimulated by circACTN4 to enhance ICC proliferation and invasion through interacting with miR-424-5p and binding to the Y-box binding protein1 [11]. Nevertheless, the gene regulation of circRNAs causing phenotypic changes in ICC remains elusive.

RNA modifications play critical roles in regulating gene expression and functions. As the most abundant internal modification of coding and non-coding RNA polymerase II transcripts, N6-methyladenosine (m6A) modification can control any aspect of mRNA post-transcriptional regulation, including splicing, export, stability, and translation, is closely associated with a variety of human diseases, particularly cancer, including ICC [12]. A recent example is that METTL16 regulated PRDM15 protein expression via YTHDF1-dependent translation to promote ICC growth [13]. Another report demonstrated that METTL3 transcription promotes ICC progression by YTHDF2-mediated IFIT2 mRNA degradation [14]. Furthermore, growing evidence suggests that m6A modification is also present in ncRNAs including circNRAs [15]. For example, N6-methyladenosine-modified circPLPP4 sustains cisplatin resistance in ovarian cancer cells via PIK3R1 upregulation [16]. ALKBH5-mediated m6A modification of circCCDC134 facilitates cervical cancer metastasis by enhancing HIF1A transcription [17]. Nevertheless, whether m6A modification of circRNAs could regulate ICC progression remains unclear.

Herein, we firstly discovered m6A-modified circSLCO1B3 as a tumor promoter in ICC. It has the potential to efficiently facilitate the proliferation, metastasis and immune evasion of ICC by regulating the expression of HOXC8 and PD-L1, indicating its significance as a crucial therapeutic target for ICC.

## Material and methods

### Patients

CircRNAs sequencing was performed by Shanghai Aksomics Technology on five pairs of ICC tissues and their respective neighboring normal tissues. The tissue samples were obtained from surgical specimens after hepatectomy at the First Affiliated Hospital of Zhengzhou University. Table S1 in Additional file 1 showed demographic characteristics of the patients who provided tissues for sequencing. ICC (*n*=13) tissues and the corresponding control normal tissues were collected from individuals who had not undergone any cancer treatment before surgical removal. The samples were rapidly frozen in liquid nitrogen and stored there until RNA extraction.

We also employed two paraffin-embedded tissue sections to test gene expression. One contains 93 paired ICC and corresponding normal tissues to detect the expression of circSLCO1B3 and HOXC8. Another contains 55 ICC tissues to detect the expression of circSLCO1B3 and PD-L1. Two pathologists independently validated each clinicopathological diagnosis. The First Affiliated Hospital of Zhengzhou University's Ethics Committee granted approval for the study (2019-KY-383) and all participants willingly signed their informed consent.

### Cell Culture

The Human ICC cell lines (RBE, CCLP1, and HCCC) and the Human normal intrahepatic bile vessel cell line HIBEpic were provided by the Type Culture Collection of the Chinese Academy of Science. HuCCT1 was obtained from Shenzhen Haodihuatuo Biotechnology Co. Ltd. All of cells were cultured in 1640 medium (Sigma, USA) supplemented with 10% fetal bovine serum (Gemini, USA), 1% streptomycin, and 1% penicillin (Solarbio, China) at a temperature of 37 °C and 5% CO_2_ in a saturated humidity atmosphere.

### Extraction of total RNA and reverse transcription, extraction of genomic DNA (gDNA), and analysis of quantitative real-time PCR (qPCR)

Total RNA was extracted by RNAiso Plus Reagent (Takala, China) from ICC cells and tissues following the instructions of manufacturer. The NanoDrop One ultramicro UV spectrophotometer was utilized to evaluate the integrity and purity of the isolated RNA. Afterward, cDNA was synthesized from 1 μg of total RNA using the PrimeScript RT Reagent Kit (Accurate Biology, China) in qPCR instrument at a temperature of 37 ℃ for 2minutes and 85 ℃ for 5 seconds. The gDNA was extracted using gDNA Reagent Kit (Sangon Biotech). The expression levels of the candidate genes were assessed using qPCR on an ABI 7900HT System (Applied Biosystems, USA). Using 2^−△△CT^ method, the expression levels of circRNAs, mRNAs and miRNAs expression levels were normalized to the reference genes GAPDH and U6. The primers sequences were listed in Additional file 1: Table S2.

### Actinomycin D assay

RBE cells and cells HuCCT1 were treated with actinomycin D (Merck, Darmstadt, Germany) at a concentration of 100ng/ml. After that, total RNA was extracted from the collected cells. qPCR was used to assess the stability of circSLCO1B3 and SLCO1B3 via constructing degradation curve.

### RNase R treatment

Total RNA (10ug/group) from HuCCT1 cells was incubated for 0, 7, 14, and 21 minutes whereas total RNA from RBE cells was treated for 0, 10, 20, and 30 minutes using RNase R (5U/ug; Epicentre Technologies, Madison, USA) at 37 ℃. Subsequently, qPCR and nucleic acid electrophoresis were carried out to quantify linear RNA and circular RNA.

### Cell transfection, plasmids and oligonucleotides

Small interfering RNAs (siRNAs) targeted mRNA and the back-splice junction site of circSLCO1B3 along with miR-502-5p mimics and inhibitors were obtained from RiboBio in Guangzhou, China. In order to strengthen gene knock-down efficiency, we merged siRNA #2 and siRNA #3 (2 μg) in RMPI 1640 media to conduct experiment. The qPCR was used to assess the efficiency of these siRNAs. Lipofectamine 3000 from Invitrogen (Carlsbad, USA) was employed for cell transfections. Table S3 in Additional file 1 contains the specific oligonucleotide sequences.

To establish stable cell lines with overexpressed circSLCO1B3, the circSLCO1B3 overexpression vector and control vector, together with the pMD2G envelope plasmid and pSPAX2 packaging plasmid, were transfected into HuCCT1 cells using Lipofiter TM (Hanbio Biotechnology). Following a 48-hour lentivirus transduction, HuCCT1 cells were exposed to puromycin (Sangon Biotech, Shanghai, China) at a concentration of 3 g/ml for a duration of two weeks in order to filtrate stable cell lines.

### Nucleic acid electrophoresis

The qPCR products of the cDNA or gDNA were visual using 3% agarose gel electrophoresis. DNA was separated using electrophoresis at 120 V for 25 minutes. The bands were exposed to UV rays to be analyzed.

### Western blotting

RIPA buffer was supplemented with proteinase and phosphatase inhibitors to extract the overall protein from ICC tissues or cell lines. To measure the protein concentration, the BCA reagent from Beyotime in China was utilized. Protein separation was achieved using SDS-PAGE gels, followed by transfer to a PVDF membrane (Millipore, Massachusetts, USA). Specific primary antibodies for the proteins HOXC8 (1:1000, #15448-1-AP, proteintech), SMAD3(1:1000, #9523, CST), SMAD2(1:1000, 12570-1-AP, proteintech), p-SMAD2 (1:1000, Ser465/467, CST), p-SMAD3 (1:1000, Ser423/425, CST), E-Cadherin (1:1000, 20874-1-AP, proteintech), N-Cadherin (1:1000, 22018-1-AP, proteintech), VIM (1:1000, #5741, CST), PD-L1 (1:1000, AB205921, Abcam) and GAPDH (1:10,000, Abcam, USA) were used to incubate the membranes overnight at 4 ℃. Following this, the membranes were incubated at 37 ℃ for 2 hours with secondary antibody (1:10000, Cell Signaling Technology, MA, USA). The targeted proteins were visualized using the enhanced chemiluminescence (ECL) reagent (Millipore, MA, USA). GAPDH served as the loading control.

### Extraction of nuclear and cytoplasmic components, fluorescence *in situ* hybridization (FISH) and Immunofluorescence (IF)

To quantitatively evaluate the expression of circSLCO1B3 in nuclear and cytoplasmic fractions, RNA was extracted from these two components using a PARISTM kit (Invitrogen, Thermo Fisher Scientific, Waltham, USA). FISH was conducted in RBE cells according to the given instructions to assess the spatial arrangement of circSLCO1B3 and miR-502-5p using a fluorescent in situ hybridization kit (C10910, Ribo-Bio, Guangzhou, China). To summarize, placed the cell slide at the bottom of the 24-well plate and added 6×10^4^ cells in each well until the cell confluence reaches a range of 60 and 70%. Following that, ICC cells underwent fixation, cleansing and permeabilization. An overnight hybridization procedure utilized FAM-labeled circSLCO1B3 probes and Cy3-labeled miR-502-5P. The pictures were acquired with the Zeiss LSM710 Laser Scanning Confocal Microscope. Similarly, the FISH test was also performed on a tissue microarray (TMA), where tissue sections were exposed to a particular FAM-labeled circSLCO1B3 probes at 37 ℃ overnight after prehybridization at the same temperature for 2 hours. Following hybridization, cell nuclei were stained with DAPI for 15 minutes. Finally, images were captured using a panoramic scanner (3DHISTECH, Hungary). The Halo v3.0.311.314 analysis software employed the Indica Labs-Area Quantification FL v2.1 module to measure the intensity and area of circSLCO1B3 staining. Moreover, the expression of HOXC8 was determined through IF performed in Tissue Microarray following the FISH assay.

### Cell proliferation assays

The assessment of ICC cell proliferation was done with the colony formation assays, cell counting Kit-8 (CCK-8) assays and EdU Apollo567 assays. 96-well plates were used to planted 3 × 10^3^/well of the treated ICC cells. Following the addition of 10 μL/well of CCK-8 solution to cell culture medium, the mixture was incubated in darkness for 2 hours. Subsequently, a microplate reader (BioTek Instruments, USA) was deployed to measure the absorbance at 450 nm every 24 hours. For the EdU incorporation assay, HuCCT1 and RBE cells were placed in 96-well plates for a 24-hour culture. Following a 2-hour incubation period using 50mM EdU solution, both cell lines were fixed using 4% paraformaldehyde and sequentially sealed using Apollo Dye Solution and Hoechst 33342. To observe and measure the EdU cells, a microscope from Olympus (Tokyo, Japan) was used. For the colony formation experiment, transfected cells were sown in 6-well plates with a density of either 500 or 1000 cells per well, and then cultured for a period of 1-2 weeks. Next, the cells were treated with 4% paraformaldehyde for a duration of 30 minutes, followed by staining with a 0.5% crystal violet solution for an additional 30 minutes. Finally, the colonies were assigned numerical values and tallied.

### Cell migration and invasion assays

To evaluate the migratory and invasive abilities of ICC cells, trans-well assays and wound healing tests were conducted. Trans-well investigations were conducted using 24-well plates equipped with chamber inserts containing pores of size 8.0 μm provided by corning. The chamber inserts were either empty or filled with 2% Matrigel (BD Science, USA). After 36 hours of transfection, the cells were trypsinized and then placed in media without serum. Next, a total of 2×10^4^ cells were introduced into each well in the upper compartments. A cell nutritional attractant was utilized in the lower chamber, consisting of 600 $\mathrm{\mu l}$ medium supplemented with 20% FBS. After incubating at 37 °C for 24 or 36 hours, the cells in the upper chamber were delicately extracted with a cotton swab. To make the migrated or invaded cells visible, they were treated with 4% polyformaldehyde and stained for 30 minutes using 0.5% crystal violet. Afterwards, the migratory or invaded cells were observed and measured using a reversed microscope (Olympus, Japan) to capture five randomly chosen microscopic areas. ICC cells were cultivated in six-well plates for the intention of performing wound healing experiments. Afterwards, the cell monolayer was scraped using a pipette tip with a volume of 1000ul. Photos were taken at 0 hours, 24 hours, or 36 hours after the scrape to capture the movement of cells in 10 high-power fields.

### Dual-Luciferase reporter assays

The anticipated miR-502-5p attachment locations within the complete wild-type (WT) circSLCO1B3 sequence, the untranslated region of HOXC8, and the specified mutant circSLCO1B3 and HOXC8 sequences (Mut) were created and inserted into the psi-CHECK-2 dual-luciferase reporter vector (Hanbio Biotechnology, China) separately. The trial is conducted following the guidelines provided by the manufacturer for the Dual Luciferase Assay Kit (Hanbio Biotechnology, China). 293 T cells were transfected with plasmids following a 48-hour interval, the cells were lysed and gathered. The firefly luciferase activities were assessed by the SpectraMax M5e, a versatile enzyme label tester that measures full-wavelength, from Molecular Devices in Shanghai, China.

### RNA immunoprecipitation (RIP) assay and Methylated RNA immunoprecipitation (MeRIP) assay

The Magna RIPTM RNA-binding protein immunoprecipitation kit (Millipore, USA) was used to carry out the RIP assay in accordance with the protocol. Magnetic beads were coated with antibodies against IgG (Millipore, MA, USA), HOXC8 (Proteintech, Wuhan, China), AGO2 (Abcam, Burlingame, USA), PD-L1 (Abcam, Burlingame, USA), METTL3, and YTHDC1 (Cell Signalling Technology, MA, USA) for 30 minutes at room temperature. To capture circSLCO1B3, the cell lysate from 2×10^7^ cells was incubated with magnetic beads coated with antibodies. TRIzol was utilized to extract the entire RNA for MeRIP. In a manner similar to RIP, magnetic beads from Millipore in Massachusetts, USA were coated with 5 μg of an anti-IgG or anti-m6A antibody and incubated at room temperature for 30 minutes. Following this, 50μg of overall RNA was introduced to the beads coated with antibodies, and then incubated in immunoprecipitation buffer containing RNase inhibitor at a temperature of 4℃ for the duration of the night. Following the digestion of proteinase K, the m6A-bound RNA precipitation was completed using the RNA Clean & Concentrator kit (ZYMO research, Hangzhou). The m6A enrichment of circSLCO1B3 was assessed using qPCR and normalized to the input.

### RNA pull-down assay

The miR-502-5p probe and the circSLCO1B3 probe were all created and produced by Genepharma (Shanghai, China). The biotin-labeled probe was exposed to streptavidin magnetic beads for two hours at 37℃ in order to make probe-coated beads. Probe-coated beads were treated with HuCCT1 and RBE cell lysates for the duration of the night at 4 ℃. The beads were washed, and the extracted RNA was separated and evaluated by qPCR. Western blotting was used to evaluate the proteins that were pulled down.

### Chromatin immunoprecipitation (ChIP) assay

To evaluate the binding of HOXC8 to SMAD3 promoters, a ChIP experiment was conducted. The cells were treated with 1% formaldehyde and then glycine was added to stop the crosslinking process. After that, the chromatin was sonicated to break it into fragments ranging from 200 to 1000 bp. The lysates were incubated overnight at 4 ℃ with gentle agitation in the presence of the HOXC8 antibody (LA281-1H2, Abnova) and Magna ChIP Protein G Magnetic beads. To increase the area of the anticipated probable HOXC8 binding sites (HSEs) in the SMAD3 promoters, four primer pairs (supplementary Table S4) were created. After eluting the ChIP complexes, they were analyzed using qPCR. The data was computed using the 2^−ΔΔCT^ technique and 3% agarose gel were used to run the qPCR products.

### Extraction and cultivation of CD8^+^ T cells from peripheral blood

Healthy individuals' peripheral blood was used to purify human CD8^+^ T cells using the Ficoll kit from Sigma-Aldrich in the United States. CD8^+^ T cells were stimulated with DynabeadsTM Human T-Activator CD3/CD28 (Gibco, CA, USA) for 24 hours at 37 ℃ and 5% CO2.

### Flow cytometry (FCM) assay for cell apoptosis

ICC cells with circSLCO1B3 deficiency or circSLCO1B3 overexpression were cultured in 24-well plates for a duration of 24 hours. Following adherence of ICC cells, CD8^+^ T cells were co-cultured with them for a duration of 48 hours at a ratio of 10:1. Afterwards, the CD8^+^ T cells were gathered and labeled with FITC and PI. PI and FITC were measured using a flow cytometer (Becon Dickinson FACSCalibur, NY, USA) at different wavelengths, specifically 530 nm and 575 nm.

### ELISA

After co-incubating CD8^+^ T cells with ICC cells deficient or overexpressing circSLCO1B3 at a ratio of 10:1 for 48 hours, the supernatants were obtained. ELISA was used to test the concentration of Interleukin 10 (IL-10), Interleukin 2 (IL-2), interferon-gamma (IFNγ), or tumor necrosis factor alpha (TNFα) (Thermo Fisher, USA; 88–7025-22, 88–7316-22, 88–7346--22, 88–7346-22). The levels of IL-10, IL-2, IFNγ, or TNFα were measured by utilizing standard curves and expressed in ng/mL.

### Protein stability

The cells were treated with cycloheximide (CHX, 50 g/ml) for the specified time periods (0, 4, 8 and 12 h). After harvesting the cells, the entire proteins were gathered, and D-L1expression levels were assessed using western blot analysis.

### Ubiquitination assay

The cell cultures were treated with MG132 (10 M; C2211, Sigma-Adlrich, USA), a proteasome inhibitor that can penetrate cell membranes, for a duration of 6 hours. Following this, an immunoprecipitation lysis buffer containing phosphatase and protease inhibitors was introduced for an additional period of 30 minutes. Anti-PD-L1 antibody (ab101267, 1:500, Abcam, Cambridge, MA, USA) or IgG (ab6709, 1:500, Abcam, Cambridge, MA, USA) immunoprecipitated the lysates overnight at 4 ℃ in a rotating pattern. The assessment of PD-L1 ubiquitination was conducted using an antibody against ubiquitin (10201-2-AP, 1:1000 Proteintech, Rosemount, Minnesota, USA). The quantity of immunoprecipitated proteins was determined using western blotting.

### Tumor xenograft models

The animals were housed and fed in a pathogen-free animal room with 12-hours light-dark cycles. All animal experiments were verified by the first affiliated hospital of Zhengzhou University Committee on Animal Experiment Ethics. For the tumor growth xenograft model, 5×10^6^ ICC cells or with LV-circSLO1B3 and/or sh-HOXC8 or LV-NC suspended in 0.1 ml phosphate-buffered saline were subcutaneously injected into the right flanks of C57BL/c or BALB/c Nude mice. Mice weight and tumor volumes were assessed every two days and computed as 0.5×length×width^2^. Finally, the mice were euthanized and the tumors were extracted for further analysis.

To evaluate cell metastasis, the animals were injected with 1×10^6^ HuCCT1 cells with LV-circSLCO1B3 or LV-NC and sh-HOXC8 through the tail vein every three days for three weeks to establish lung metastasis model.

### Statistical analysis

Mean±SD was displayed for the data after analysis using GraphPad Prism 9.0 (Graph-Pad, San Diego, CA, USA). Two separate groups were compared using an unpaired Student's t-test. The log-rank (Mantel-Cox) tests were used to analyze survival curves. Based on the FISH scores of circSLCO1B3 (high or low), all patients were divided into two groups for the Kaplan-Meier analysis. A binary cutoff value was created using the best Youden's index on the receiver operating curve (ROC) of ICC progression during a 12-month period. Statistics were deemed as significant at *P* < 0.05. Software such as Image J, Adobe Photoshop 2020, and Adobe Illustrator 2020 were utilized to stitch together the images.

## Results

### CircSLCO1B3 was highly expressed and correlated with poor prognosis in ICC patients

**Fig. 1:**
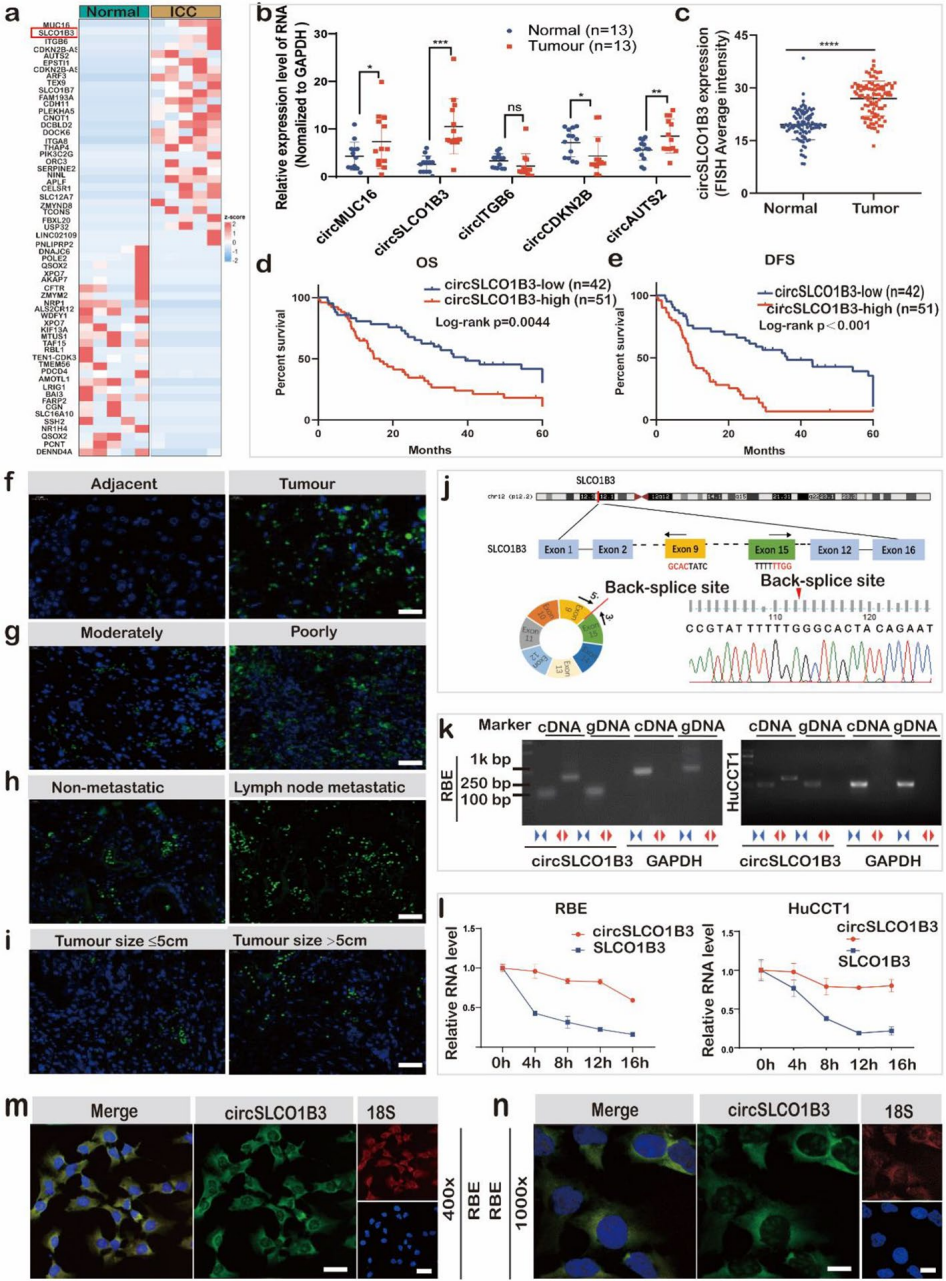
CircSLCO1B3 is highly expressed and correlated with poor prognosis in ICC patients. **a** Cluster heat map of the representative results of circRNA-seq. **b** The expression level of circRNAs with significantly high expression in CCA tissues were detected by qPCR in 13 CCA tissues and matched normal tissues. **c** FISH scores statistical analysis of circSLCO1B3 in ICC tissues and normal tissues (*n*=93). **d**,** e** Kaplan-Meier analyses of the correlation between circSLCO1B3 expression and overall survival or recurrence. **f**,** g**,** h**, **i** FISH images of circSLCO1B3 in ICC tissues and normal tissues (circSLCO1B3-FISH: green spots, DAPI: blue spots) (magnification, × 200, scale bar, 50 μm). **j** Schematic illustration of the genomic location and back splicing of circSLCO1B3, with the splicing site validated by sanger sequencing. **k** Convergent and divergent primers were employed to confirm the closed loop topology of circSLCO1B3. **l** qPCR analysis of circSLCO1B3 and linear SLCO1B3 in ICC cells treated with Actinomycin D at the indicated time point. **m**,** n** The subcellular location of circSLCO1B3 in RBE cells by FISH (DAPI: blue spots) (magnification, × 400, scale bar, 20 μm and magnification, × 1000, scale bar, 10 μm). (Values are expressed as the means ± SDs; *P < 0.05, **P < 0.01 and ***P < 0.001)

Circular RNAs are a unique class of ncRNAs that have been identified as important ICC modulators [18]. Nevertheless, the precise functions and intricate mechanisms involved in tumorigenesis remain unclear thus far. In our study, 2199 differentially expressed circRNAs transcripts were found in 5 pairs of cancer and adjacent tissues of patients with ICC via RNA-seq analysis (Fig. S1a). The volcano map revealed 83 up-regulated and 59 down-regulated circRNAs in tumor tissues of ICC patients compared to the adjacent tissues via fold change > 2, and p < 0.01 (Fig. S1b). Among them, the top 30 dysregulated circRNAs were displayed in heat map (Fig. 1a). According to GO functional annotation and KEGG pathway analysis, these differentially expressed genes were primarily grouped in the cell cycle, cell division, and immune-related pathway of ICC (Fig. S1c). Given that a vast majority of circRNAs in low abundance compared with their linear transcripts, current reports focus on highly expressed circRNAs in tumor tissues hoping to find the new therapeutic targets [19]. Thus, we focused on the top 5 exonic circRNAs with up-regulated expression, namely: circMUC16, circSLCO1B3, circITGB6, circCDKN2B-AS, circAUTS2. Then, further detected the relative expression levels of 5 circRNAs by RT-PCR in 13 paired ICC and adjacent tissues. The results showed that compared with the adjacent tissues, the expression levels of circCDKN2B-AS were down-regulated, circITGB6 was not significantly different, while the expression circMUC16, circSLCO1B3 and circAUTS2 were up-regulated, with circSLCO1B3 being the most significantly up-regulated (Fig. 1b). Therefore, we chose circSLCO1B3 as the research target.

CircSLCO1B3, which derived from exons 9-15 of the SLCO1B3 gene by back-splicing on the basis of annotation of CIRCexplorer2, also was up-regulated in ICC cells versus bile duct epithelial cell line (Fig. S1d). The predictive significance of circSLCO1B3 was assessed using FISH tests on paraffin-embedded tissue slices which contained 93 pairs of ICC and matched surrounding normal tissues. The results indicated that the expression of circSLCO1B3 was significantly elevated in ICC tissues compared with para-cancerous, which was consistent with the results of RNA-seq (Fig. 1c). According to the best Youden's index on the ROC of ICC progression within 1 year (Fig. S1e), the cohort of 93 ICC patients was segregated into two groups: high-expression performance of circSLCO1B3 (*n*=51) and low expression performance of circSLCO1B3 (*n*=42). The survival analysis illustrated that the high circSLCO1B3 expression group was relative with a poorer prognosis among patients with ICC (Fig. 1d, e). Of note, the FISH images and scores demonstrated that circSLCO1B3 was distinctly upregulated in cholangiocarcinoma versus normal tissues (Fig. 1f). Furthermore, there was a significant correlation observed between high FISH scores and tumor size, differentiation status, and lymph node metastasis (Fig. 1g, h, i, Table 1).

**Table 1:** Clinical information and circSLCO1B3 expression in ICC patients

**Parameters**	**Number of cases**	**CircSLCO1B3 expression** **(Average Intensity)**		***χ***^***2***^	***P* value**
		**Low**	**High**		
**All cases**	**93**	**42**	**51**		
**Age**				***3.587***	***0.058***
<48	**60**	**14**	**46**		
≥48	**36**	**15**	**21**		
**Gender**				**0.228**	**0.633**
Male	**60**	**26**	**34**		
Female	**33**	**16**	**17**		
**Histological differentiation**				***4.207***	***0.04***
Moderately differentiated	**38**	**22**	**16**		
Poorly differentiated	**55**	**20**	**35**		
**TNM**				**0.290**	**0.590**
TI-TII	**57**	**27**	**30**		
TIII-TIV	**36**	**15**	**21**		
**Tumour size**				***7.990***	***0.005***
≤5cm	**43**	**23**	**10**		
>5cm	**50**	**19**	**31**		
**Lymph node metastatic**				***7.168***	***0.007***
Yes	**18**	**5**	**13**		
No	75	**47**	**28**		
**CA19-9**				**0.861**	**0.353**
≤35u/ml	**40**	**18**	**22**		
>35u/ml	**53**	**29**	**24**		

Even with the increased sensitivity and accuracy of RNA-seq and algorithms, experimental validation is needed to confirm circRNA candidates identified from large-scale analyses prior to carrying out characterization and functional studies. Therefore, the ring structure of circSLCO1B3 was further determined through various methods, including sanger sequencing, verification using divergent and convergent primers, actinomycin D assay and RNase R assay. The result of sanger sequence was consistent with RNA-seq, which showed that the qPCR products amplified using a divergent primer contained the sequence of back-splice junctions (Fig. 1j). In addition, amplification of post-transcriptional circSLCO1B3 was only observed with divergent primers using cDNA rather than gDNA as a template (Fig. 1k). Furthermore, RNase R assay found that circSLCO1B3 was more resistant to digestion by exonuclease than linear SLCO1B3 and GAPDH (Fig. S1f, g). Meanwhile, actinomycin D was utilized to stop gene transcription, and circSLCO1B3 was relatively more stable than linear form (Fig. 1l). These results proved that circSLCO1B3 was a ring structure. And beyond that, the result of nuclear and cytoplasmic separation experiment indicated that circSLCO1B3 exhibited a predominant localization within the cytoplasm of RBE and HuCCT1 cells (Fig. S1h). FISH assay also showed that the majority of circSLCO1B3 (green) was localized within the cytoplasm of RBE cells (Fig. 1m, n).

### CircSLCO1B3 promoted proliferation, migration and invasion of ICC cells *in vitro*

**Fig. 2:**
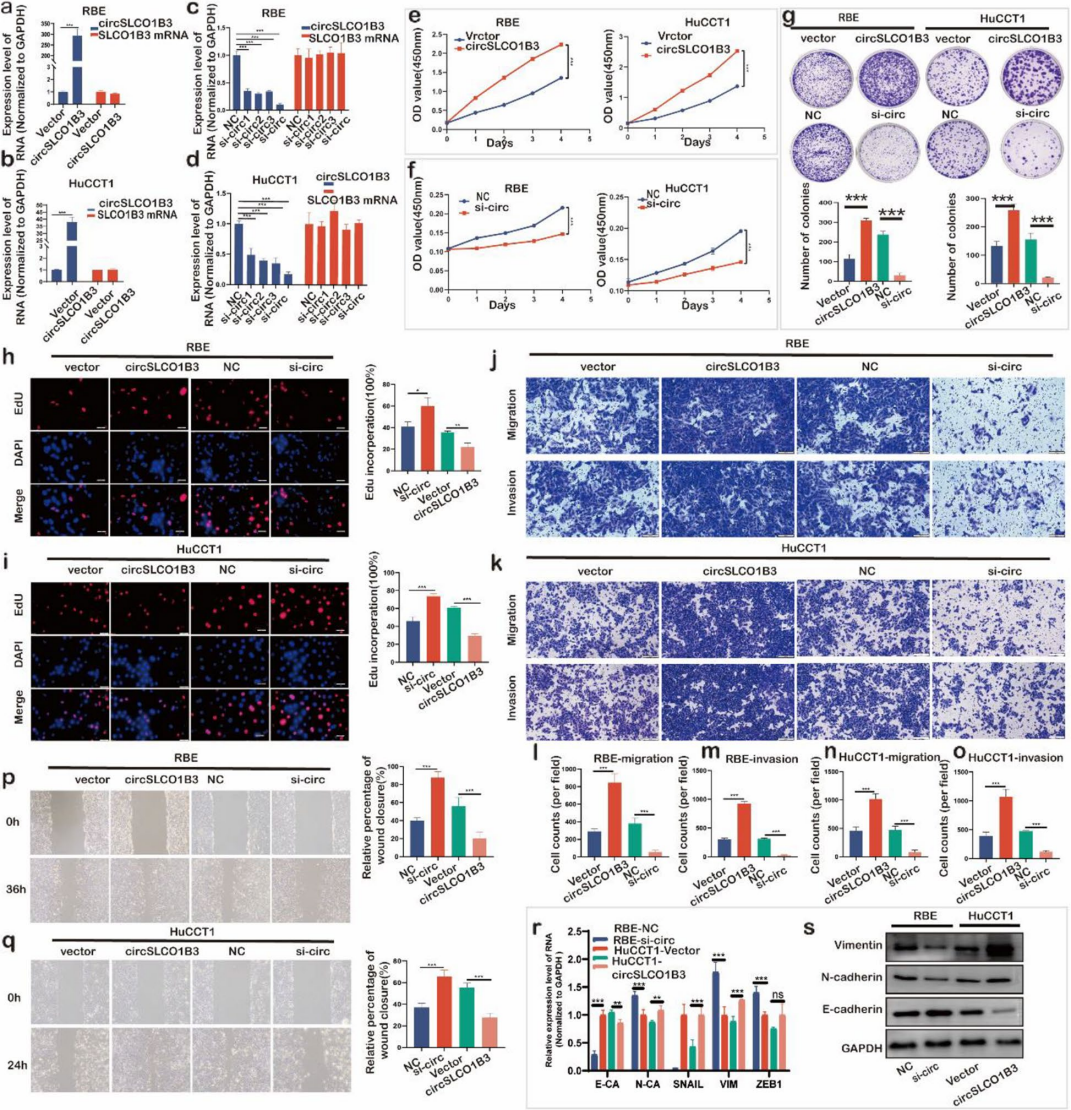
CircSLCO1B3 promoted proliferation, migration and invasion of ICC cells in vitro. **a**,** b**,** c**,** d** qPCR analysis of circSLCO1B3 and linear SLCO1B3 expression in ICC cells transfected with circSLCO1B3 siRNAs or plasmids. **e**,** f** The growth curves of ICC cells were evaluated by CCK-8 assays. **g** The colony formation assays showed the cell colony number after knocking down and overexpressing circSLCO1B3 in CCA cells. **h**, **i** EdU assays of CCA cells were performed to evaluate cell proliferation (magnification, × 200, scale bar, 100μm). **j**,** k**,** l**,** m**,** n**,** o**,** p**,** q** Trans-well assays and wound healing assays were conducted to determined cell migration and invasion (magnification, × 200, scale bar, 100 μm). **r**,** s** qPCR and western blotting assays to examine the effect of circSLCO1B3 on epithelial and mesenchymal marker expression. Data were showed as mean ± SD. *P < 0.05, **P < 0.01, ***P < 0.001

To explore circSLCO1B3^’^s biological function, RBE and HuCCT1 were chosen for overexpression and knockdown experiments (Fig. S2a, b). CircSLCO1B3 was effectively overexpressed by transfection of the circular transcript expression vector circSLCO1B3 in ICC cells without altering the expression of SLCO1B3 mRNA (Fig. 2a, b). Meanwhile, it was potently repressed by siRNA #2 and/or #3, according to the qPCR data, with no effect on the expression of its host SLCO1B3 mRNA (Fig. 2c, d). We chose si-circSLCO1B3-2+3 (hereafter referred to as si-circ) for deleting the circSLCO1B3 due to its high inhibitory efficiency. In CCK8 experiments, circSLCO1B3 overexpression clearly increased ICC cell proliferation, whereas circSLCO1B3 knockdown showed the opposite effect (Fig. 2e, f). Additionally, the colony formation assays demonstrated that circSLCO1B3 overexpression greatly increased while circSLCO1B3 knockdown significantly decreased the number of cell colonies (Fig. 2g). Similarly, the percentage of EdU-positive cells fluctuated high with the overexpression of circSLCO1B3, but low significantly with the knockdown of circSLCO1B3 (Fig. 2h, i). As a result, these experiments suggest that circSLCO1B3 promotes proliferation in ICC cells.

Then, further studies were conducted using trans-well and wound healing assays to evaluate how circSLCO1B3 affects migration and invasion. In Fig 2j, k, l, m, n, o, p, q, RBE and HuCCT1 showed markedly increased or decreased migratory and invasive capabilities by upregulating or downregulating circSLCO1B3, respectively. Intriguingly, when circSLCO1B3 was overexpression in HuCCT1, the cell morphology changed from typical cobblestone-like appearance to a spindle-shaped or elongated mesenchymal form (Fig. S2c). Whereas RBE cells with depleting circSLCO1B3 tended to exhibited typical cobblestone-like appearance (Fig. S2d). These findings imply that circSLCO1B3 may significantly contribute to the epithelial-mesenchymal transition (EMT) of ICC cells. Then, the impact of circSLCO1B3 on the expression of epithelial and mesenchymal markers was investigated. The results demonstrated that there was a remarkable rise in the expression of E-cadherin, but a drop in the expression of the N-cadherin and Vimentin in HuCCT1 cells with downregulation of circSLCO1B3. In contrast, circSLCO1B3 upregulation showed the opposite results in RBE cells (Fig 2r, s). Collectively, these findings indicated that circSLCO1B3 induces the migration, invasion, and epithelial-mesenchymal transition of ICC cells.

### CircSLCO1B3 promoted ICC progression via inhibiting expression of miR-502-5p

**Fig. 3:**
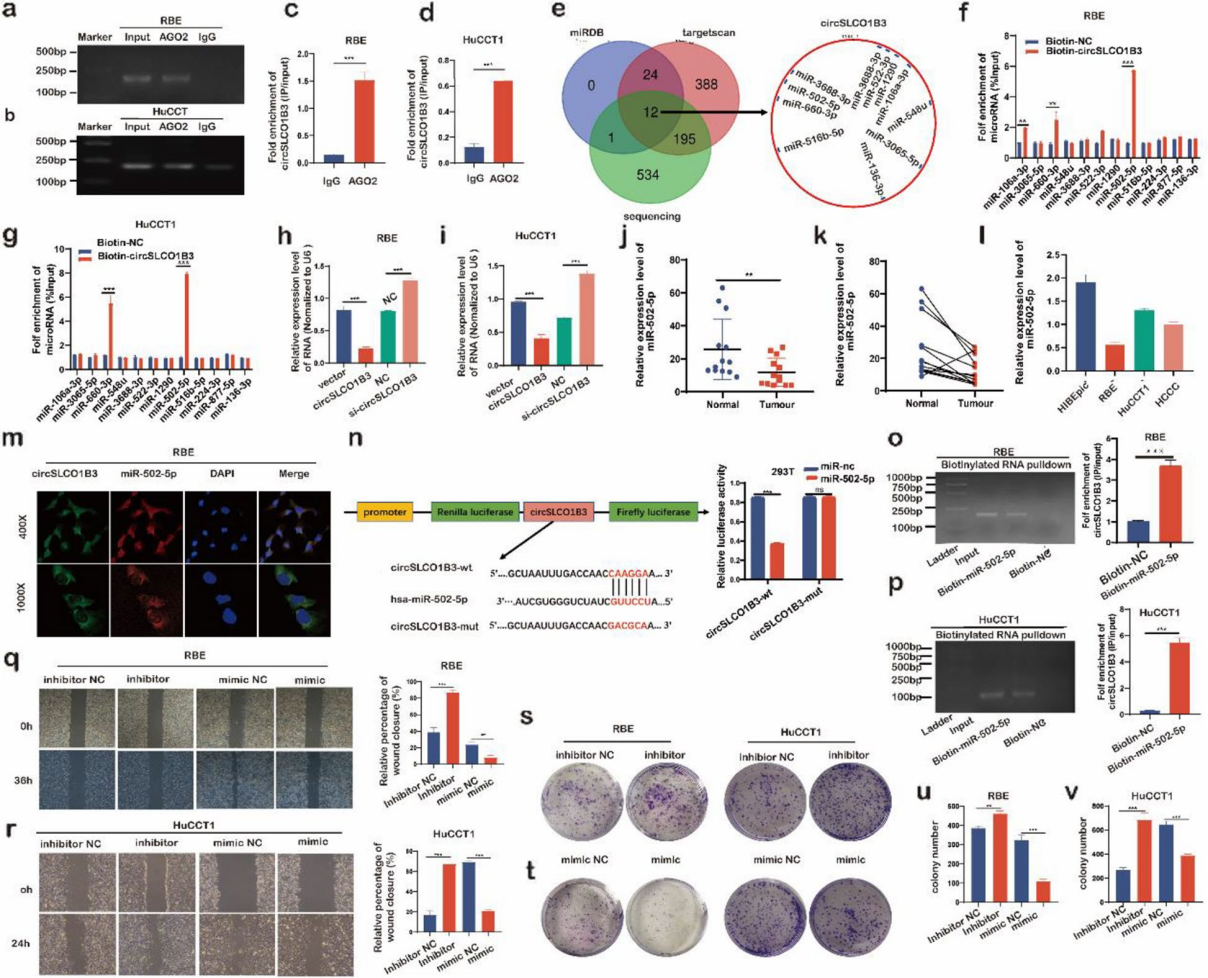
CircSLCO1B3 promoted ICC progression via inhibiting expression of miR-502-5p. **a**,** b**,** c**,** d** RIP assays using an anti-Ago2 antibody and IgG were performed in RBE and HuCCT1 cells. **e** Schematic illustration exhibiting overlapping of the target miRNAs of circSLCO1B3 predicted by miRDB database, targetscan database and microRNA-seq results. **f**,** g** RNA pull-down was executed in RBE and HuCCT1 cells to determine the relative expression levels of 12 potential target miRNAs by the circSLCO1B3 probe. **h**, **i** qPCR evaluated the expression of miR-502-5p after orchestrating circSLCO1B3 in ICC cells. **j**,** k**,** l** qPCR determined the expression of miR-502-5p in ICC tissues and cells. **m** The cellular locations of circSLCO1B3 (green) and miR-502-5p (red) in RBE cells were examined using FISH assays (magnification, × 400, scale bar, 20 μm and magnification, × 1000, scale bar, 10 μm). **n** Schematic illustrations and the relative luciferase activities of circSLCO1B3-WT and circSLCO1B3-MUT luciferase reporter vectors. **o**,** p** Pull-down was executed in ICC cells to detect the enrichment of circSLCO1B3 by the miR-502-5p probe.** q**,** r**,** s**,** t**,** u**,** v** Wound healing assays and colony formation assays were conducted to determine the migratory and proliferative capabilities in ICC cells transfected with miR-502-5p mimics and inhibitors. Data were showed as mean ± SD. *P < 0.05, **P < 0.01, ***P < 0.001

The cytoplasmic localization and remarkable stability of circSLCO1B3 piqued our interest in its potential involvement in promoting malignant biological behaviors in ICC cells through miRNA targeting. To this end, RIP assays were conducted and the findings indicated that the endogenous circSLCO1B3 was successfully pulled down by the anti-Ago2 antibody, which suggested the potential of circSLCO1B3 binding miRNAs (Fig. 3a, b, c, d). Then, miRDB database, targetscan database and microRNA-seq results were used and overlapped to predicted the potential targets of circSLCO1B3. 12 candidate miRNAs were selected for further validation (Fig. 3e). The biotin probes were designed to target the back splicing junction of circSLCO1B3 to conduct circRNAs pull down assay. The findings showed that miR-502-5p enrichment levels were the highest of all miRNAs in both ICC cell lines (Fig. 3f, g). qPCR data revealed that miR-502-5p levels were considerably higher in circSLCO1B3-depleted ICC cells and significantly lower in circSLCO1B3-overexpressed ICC cells (Fig. 3h, i). Meanwhile, miR-502-5p was markedly downregulated in 13 pairs of ICC tissues and ICC cell lines versus adjacent non-tumor tissues and biliary epithelial cell, respectively (Fig. 3j, k, l). Furthermore, confocal imaging showed that circSLCO1B3 (green) and miR-30c-5p(red) were chiefly accumulated in the cytoplasm of RBE cells (Fig. 3m).

Dual-luciferase reporter assay was conducted to verify the relationship between circSLCO1B3 and miR-502-5p. The results revealed a noteworthy reduction in luciferase activity within the circSLCO1B3-wild group upon introduction of miR-502-5p mimics, while no such effect was observed in the circSLCO1B3-mutant group. These results strongly imply a direct interaction between miR-502-5p and circSLCO1B3 (Fig. 3n). Coincidentally, a microRNA pull-down assay suggested that a great amount of circSLCO1B3 was pulled down in the miR-502-5p probe group (Fig. 3o, p). The above results prompted us that circSLCO1B3 could directly bind with miR-502-5p to suppress its expression in ICC cells. The functional significance of miR-502-5p in ICC was further examined through colony formation assays and wound healing assays. As the results suggested, the transfection of miR-502-5p inhibitor gave rise to the proliferative and migratory abilities of ICC cells significantly, whereas transfection with miR-502-5p mimics resulted in their suppression (Fig. 3q, r, s, t, u, v). Taken together, our findings imply that circSLCO1B3 may strengthen cholangiocarcinoma cell growth and invasion by suppressing miR-502-5p expression in ICC cells.

### CircSLCO1B3 regulated miR-502-5p/HOXC8/SMAD3 axis to promote proliferation, migration and invasion in ICC

**Fig. 4:**
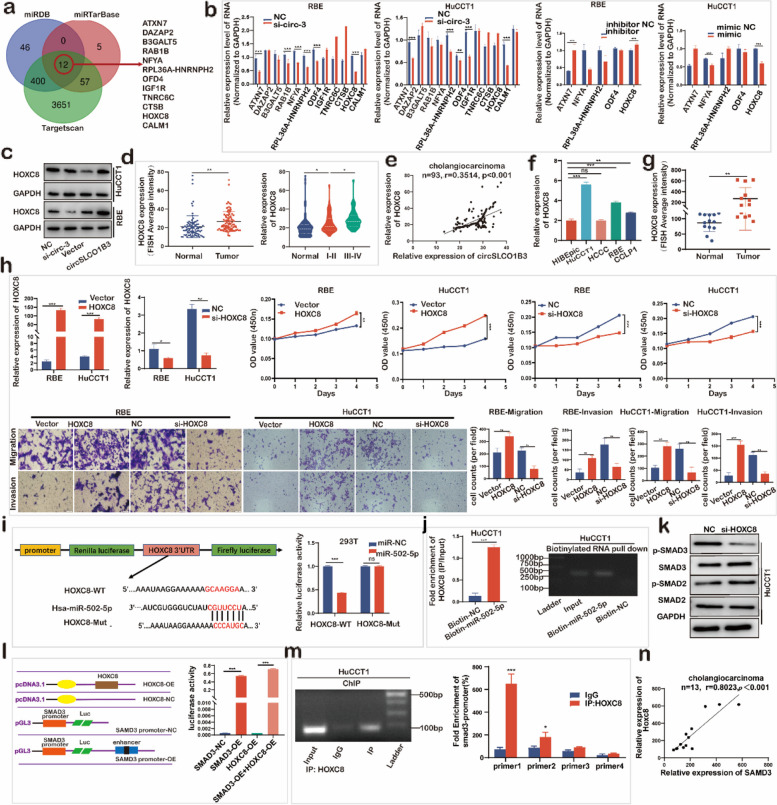
CircSLCO1B3 regulates miR-502-5p/HOXC8/SMAD3 axis to promote proliferation, migration and invasion in ICC. **a** Schematic illustration exhibited overlapping of the target mRNAs of miR-502-5p predicted by miRDB, miRTarBase and TargetScan databases. **b** qPCR was conducted to determine the relative expression levels of 12 potential target mRNAs after downregulation of circSLCO1B3 or miR-502-5p and upregulation of miR-502-5p in ICC cells. **c** Western blotting was performed to determine the expression of HOXC8 in ICC cells after overexpression or downregulation of circSLCO1B3. **d** FISH scores statistical analysis of circSLCO1B3 in ICC tissues and normal tissues. **e** The correlation of HOXC8 and circSLCO1B3 in FISH and IF scores. **f**,** g** qPCR showed that the expression of HOXC8 in ICC cell lines and tissues. **h** CCK-8 assays and trans-well assays were performed to determine the ability of proliferation and migration in ICC cells transfected with HOXC8 vectors and HOXC8 siRNA (magnification, × 200, scale bar, 100 μm). **i** Schematic illustration and the relative luciferase activities of HOXC8-WT and HOXC8-Mut luciferase reporter vectors. **j** RNA pull-down assay was executed in HuCCT1 cells to detect the enrichment of HOXC8 by the miR-502-5p probe. **k** Western blotting was performed to determine the expression of smad2, p-smad2, smad3, p-smad3 in HuCCT1 cells after depleting circSLCO1B3. **l** Luciferase plasmids with the first 2,200 nucleotides of SMAD3 promoter domain or with blank plasmids were formed to detect the associative relation of SMAD3 and HOXC8. **m** ChIP assays were executed to detect the relation of SMAD3 promoter domain and HOXC8. **n** qPCR was conducted to detect the expression relation of HOXC8 and SMAD3 in 13 pairs ICC tissues and normal tissues. Data were showed as mean ± SD. *P < 0.05, **P < 0.01, ***P < 0.001

By comparing the miRNA target predictions from miRDB, miRTarBase, and TargetScan databases, we found a total of 12 potential mRNAs that could be targeted by miR-502-5p (Fig. 4a). In the si-circSLCO1B3 and miR-502-5p mimics group, the qPCR findings indicated a reduction only in the HOXC8 expression, whereas in the miR-502-5p inhibitor group, an elevation was observed (Fig. 4b). Meanwhile, the protein level of HOXC8 rose as the circSLCO1B3 overexpressed and dropped as the circSLCO1B3 was downregulated (Fig. 4c). Moreover, FISH assays using tissue array indicated that the expression of HOXC8 was considerably upregulated in ICC tissues and markedly increased as the TNM grade goes up (Fig. 4d). HOXC8 and circSLCO1B3 showed potential of positive correlation in FISH scores (Fig. 4e). Meanwhile, qPCR data showed that the expression of HOXC8 in ICC cell lines and ICC tissues turned out to be remarkably higher compared with HIBEpic cells and adjacent normal tissues (Fig. 4f, g). Additionally, CCK8 assays and trans-well assays demonstrated that the proliferation, migration, and invasion abilities of ICC cells were distinctly enhanced via up-regulating HOXC8 and suppressed via exhausting HOXC8 (Fig. 4h).

Luciferase assays were constructed, and the results confirmed that MiR-502-5p directly targeted HOXC8 in ICC cells (Fig. 4i). Coincidentally, RNA pull-down assays indicated that the specific enrichment of HOXC8 was more significant on the miR-502-5p probe than control probe group (Fig. 4j). As shown above, miR-502-5p was designed to target HOXC8, therefore through miR-502-5p/HOXC8 axis, circSLCO1B3 could possibly promote ICC progression.

Considering that HOXC8 acts as a transcription factor and is reported in tumorigenesis and metastasis via TGF-ß signaling [20]. Therefore, we examined expression of SMAD2, p-SMAD2, SMAD3, p-SMAD3 and found that inhibiting HOXC8 decreased smad3 and p-smad3 in HuCCT1 cells (Fig. 4k). According to the Jaspar database, four HOXC8 binding sites were predicted in the SMAD3 promoter. Dual-Luciferase Reporter Assay showed that SMAD3-pGL3-wt had promoter activity and HOXC8 could affect SMAD3-pGL3-wt luciferase activity (Fig. 4l). Furthermore, ChIP assays also showed that anti-HOXC8 antibody highly enriched SMAD3 promoter fragments in comparison to IgG antibody (Fig. 4m). In addition, the expression of HOXC8 and SMAD3 was identified a positive relationship (Fig. 4n). Thus, we concluded that HOXC8 acts as the direct target of miR-502-5p and could transcriptionally regulate SMAD3 expression to activate TGF-ß/SMAD pathway in ICC cells. Taken together, circSLCO1B3 could promote the proliferation, migration and invasion of ICC via the miR-502-5p/HOXC8/SMAD3 axis.

### CircSLCO1B3 depended on HOXC8 to regulated cholangiocarcinoma growth

**Fig. 5:**
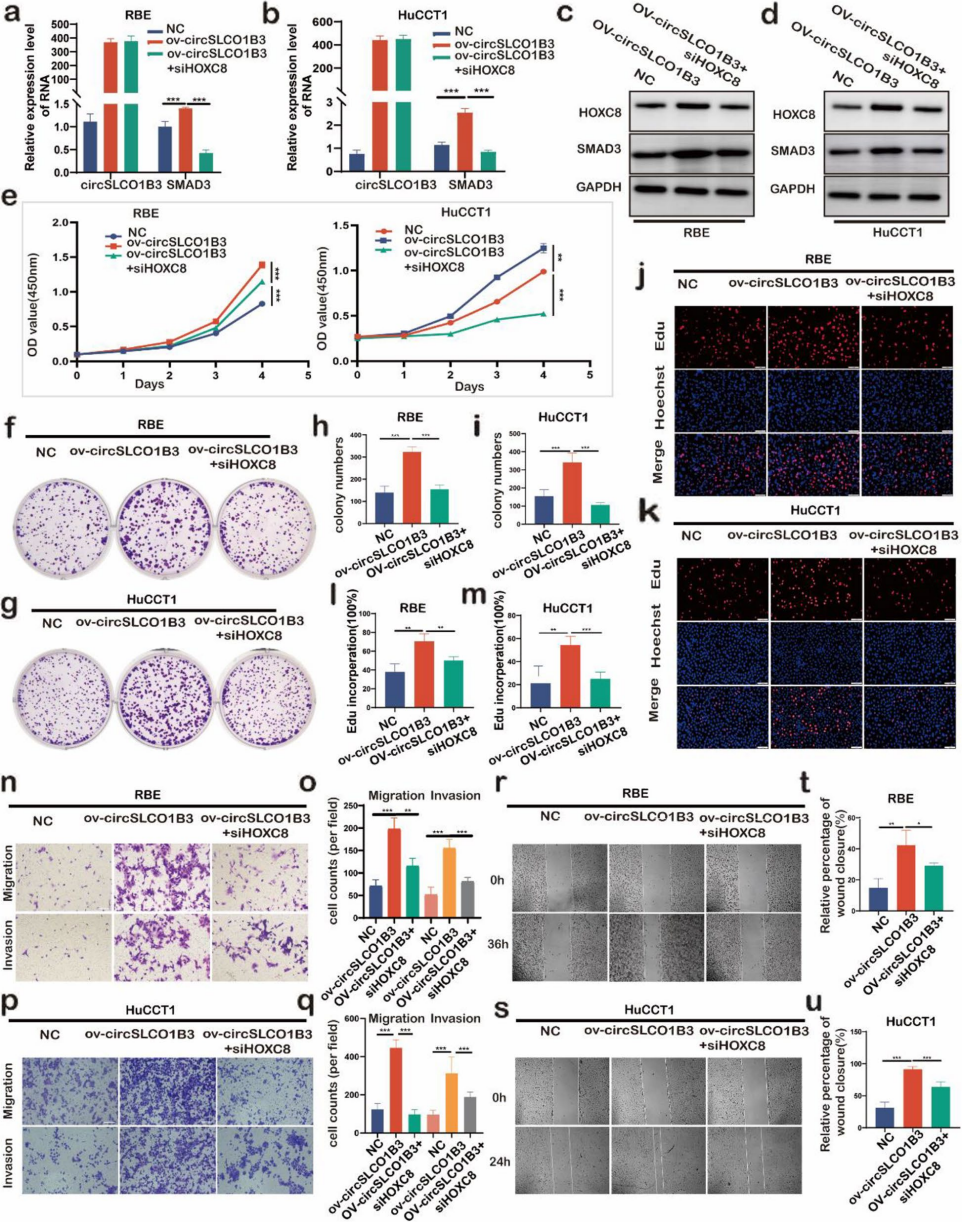
CircSLCO1B3 regulated cholangiocarcinoma growth depend on HOXC8. **a**,** b**,** c**,** d** Relative mRNA and protein levels of circSLCO1B3, HOXC8 and SMAD3 in ICC cells transfected with overexpression circSLCO1B3 vectors and HOXC8 siRNA were assessed by qPCR and western blotting. **e**,** f**,** g**,** h**, **i**,** j**,** k**,** l**,** m** CCK-8 assays, colony formation assays and EdU assays were performed to determine the ability of proliferation in RBE and HuCCT1 cells transfected with circSLCO1B3 vectors and HOXC8 siRNA (magnification, × 200, scale bar, 100 μm). **n**,** o**,** p**,** q**,** r**,** s**,** t**,** u** Trans-well assays and wound healing assays were conducted to detect the migratory and invasive capabilities in RBE and HuCCT1 cells transfected with circSLCO1B3 vectors and HOXC8 siRNA (magnification, × 200, scale bar, 100 μm). Data were showed as mean ± SD. *P < 0.05, **P < 0.01, ***P < 0.001

To explore the crucial function of HOXC8 in the promotion of ICC progression by circSLCO1B3, rescue experiments were executed via simultaneously over-expressing circSLCO1B3 and depleting HOXC8 in ICC cells. qPCR and western blotting substantiated the upregulating function of circSLCO1B3 in elevating the expression of HOXC8 and SMAD3 in ICC cells. Depleting the HOXC8 could rescued the expression of protein SMAD3 (Fig. 5a, b, c, d). Besides, we also investigated if the biological role of circSLCO1B3 could also be reversed by ablating HOXC8 in ICC cells. CCK8 assay, colony formation assay and EdU assay demonstrated an enhanced growth ability of RBE and HuCCT1 cells treated with circSLCO1B3 overexpressed plasmid compared with ICC cells treated with mock plasmid, then the increasing trend was suppressed by ablating HOXC8 (Fig. 5e, f, g, h, i, j, l, m). A similar result of trans-well assay and wound healing assay revealed that ICC cells treated with circSLCO1B3 overexpressed plasmid were observed to have a stronger migration and invasion abilities than those with mock plasmid. However, the increasing trend was suppressed by ablating HOXC8 (Fig. 5n, o, p, q, r, s, t, u). Collectively, these results indicated that circSLCO1B3 induced ICC cells proliferation, migration and invasion depending on HOXC8.

### METTL3-mediated m6A modification enhanced circSLCO1B3 stability via YTHDC1

**Fig. 6:**
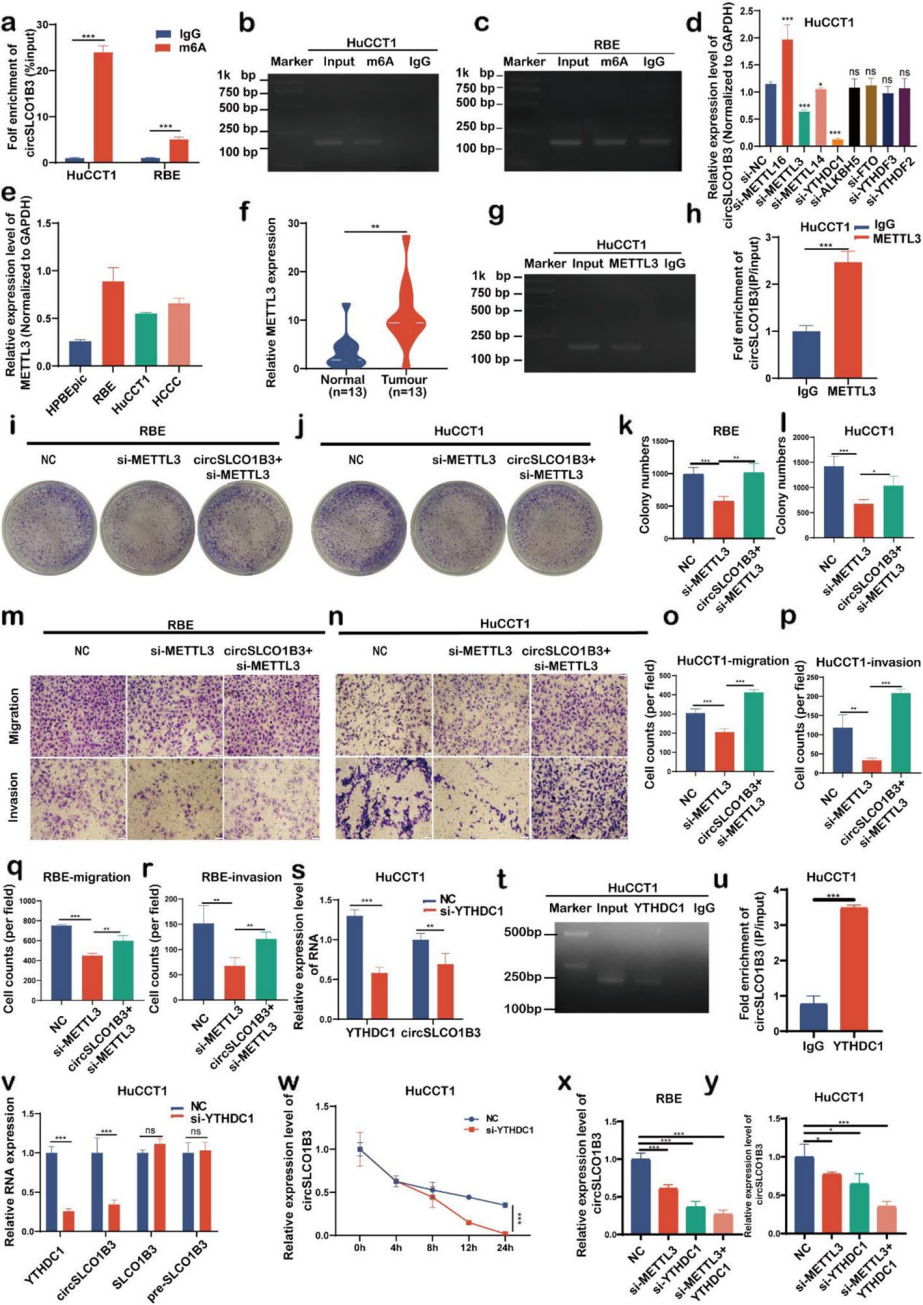
METTL3-mediated m6A modification enhanced circSLCO1B3 stability via YTHDC1. **a**,** b**,** c** MeRIP analysis of the abundance of m6A-modified circSLCO1B3 in RBE and HuCCT1 cells. **d** qPCR was performed to determine the key regulator of m6A modification in circSLCO1B3 regulation. **e**,** f**, qPCR was performed to determine the expression of methyltransferase METTL3 in CCA cells and tissues. **g**,** h** RIP assays were executed to explore the enrichment of circSLCO1B3 by the anti-METTL3 antibody. **i**,** j**,** k**,** l** Colony formation assays were performed to determine the ability of proliferation in RBE and HuCCT1 cells transfected with METTL3 siRNA and circSLCO1B3 vectors. **m**,** n**,** o**,** p**,** q**,** r** Transwell assays were conducted to detect the migratory and invasive capabilities in RBE and HuCCT1 cells transfected with METTL3 siRNA and circSLCO1B3 vectors (magnification, × 200, scale bar, 100 μm). **s** qPCR was conducted to detect the circSLCO1B3 expression levels in HuCCT1 cells transfected with YTHDC1 siRNA. **t**,** u** RIP assays were executed to explore the enrichment of circSLCO1B3 by the anti-YTHDC1 antibody. **v** Relative RNA levels of circSLCO1B3, SLCO1B3, and pre-SLCO1B3 upon YTHDC1 knockdown in HuCCT1 cells. **w** Actinomycin D RNA stability assays were conducted in HuCCT1 after transfecting with YTHDC1 siRNA. **x**,** y** The expression levels of circSLCO1B3 after simultaneously transfecting with YTHDC1 and METTL3 siRNA. Data were showed as mean ± SD. *P < 0.05, **P < 0.01, ***P < 0.001

M6A-modification has the greatest impact on circRNAs dynamic regulation and is critical for circRNAs nuclear transport, degradation, back-splicing and stability [21]. To verified whether m6A can modulate circSLCO1B3, we searched the online website SPAMP and found two m6A sites (Fig. S3a). Next, according to the results of MeRIP, the circSLCO1B3 was selectively enriched by anti-m6A antibodies in RBE cells and was found to be more densely in HuCCT1 cells with high circSLCO1B3 expression (Fig. 6a, b, c). These findings showed that circSLCO1B3 has the m6A modification, which may have potential importance for circSLCO1B3 production.

In order to ascertain the key influential factor of m6A modification regulation, we silenced METTL3, METTL14, METTL16, FTO, ALKBH5, YTHDF1, YTHDF2 and YTHDC1 separately, and found that circSLCO1B3 was significantly down-expressed in HuCCT1 cells when we transfected with si-METTL3 and si-YTHDC1 (Fig. 6d). As an oncogene, METTL3 reportedly catalyzed the modification of target genes by m6A in ICC (23). In our study, METTL3 was shown to be considerably up-regulated in ICC cells, GEPIA2 dataset, and our dataset (Fig. 6e, f, Fig. S3b). Meanwhile, the qPCR data indicated a positive association between METTL3 expression and circSLCO1B3 expression in ICC tissues (Fig. S3c). An RIP assay validated the interaction between circSLCO1B3 and METTL3. Furthermore, METTL3 antibodies exhibited a substantially higher affinity for circSLCO1B3 compare to IgG antibodies via pull-down assay (Fig. 6g, h).

To explore the role of METTL3 in ICC cells, cell functional experiments were carried out. The results showed that the colony number and migration and invasion capabilities of ICC cells were greatly attenuated by knockdown of METTL3 and reversed by upregulation of circSLCO1B3 (Fig. 6i, j, k, l, m, n, o, p, q, r). Based on these findings, METTL3-mediatedm6A modification modulates ICC progression by altering circSLCO1B3 expression.

YTH domain containing 1 (YTHDC1) is an m6A reader and has been shown that can regulate circRNAs back-splicing. We firstly found that depletion of circSLCO1B3 could decreased the expression of YTHDC1 and YTHDC1 antibodies could pull down the circSLCO1B3 (Fig. 6s, t, u). We then designed primers for pre-SLCO1B3 and performed qPCR. The results indicated that YTHDC1 silencing in HuCCT1 cells significantly decreased circSLCO1B3 expression, whereas its precusor transcript and linear SLCO1B3 mRNA remained unchanged (Fig. 6v). Therefore, we ruled out the possibility that YTHDC1 affects the back-splicing of circRNAs. In view of the fact that m6A modification also influences the stability of RNAs, actinomycin D RNA stability assays were conducted in HuCCT1 after transfecting with YTHDC1 siRNA, qPCR analysis of circSLCO1B3 was done at 0, 4, 8, 12, 24h. The results indicated that the half-life of circRNAs rapidly decreased in YTHDC1 siRNA group compared with NC siRNA group (Fig. 6w). These results suggested that YTHDC1 may regulate m6A-mediated circSLCO1B3 by facilitating its stability. Moreover, qPCR demonstrated that the expression of circSLCO1B3 after simultaneously transfecting with YTHDC1 and METTL3 siRNA was lower than separately silencing one (Fig. 6x, y). Taken together, METTL3-mediated m6A modification strengthened circSLCO1B3 stability via YTHDC1 to enhancing ICC progression.

### CircSLCO1B3 overexpression promoted cholangiocarcinoma progression *in vivo*

**Fig. 7:**
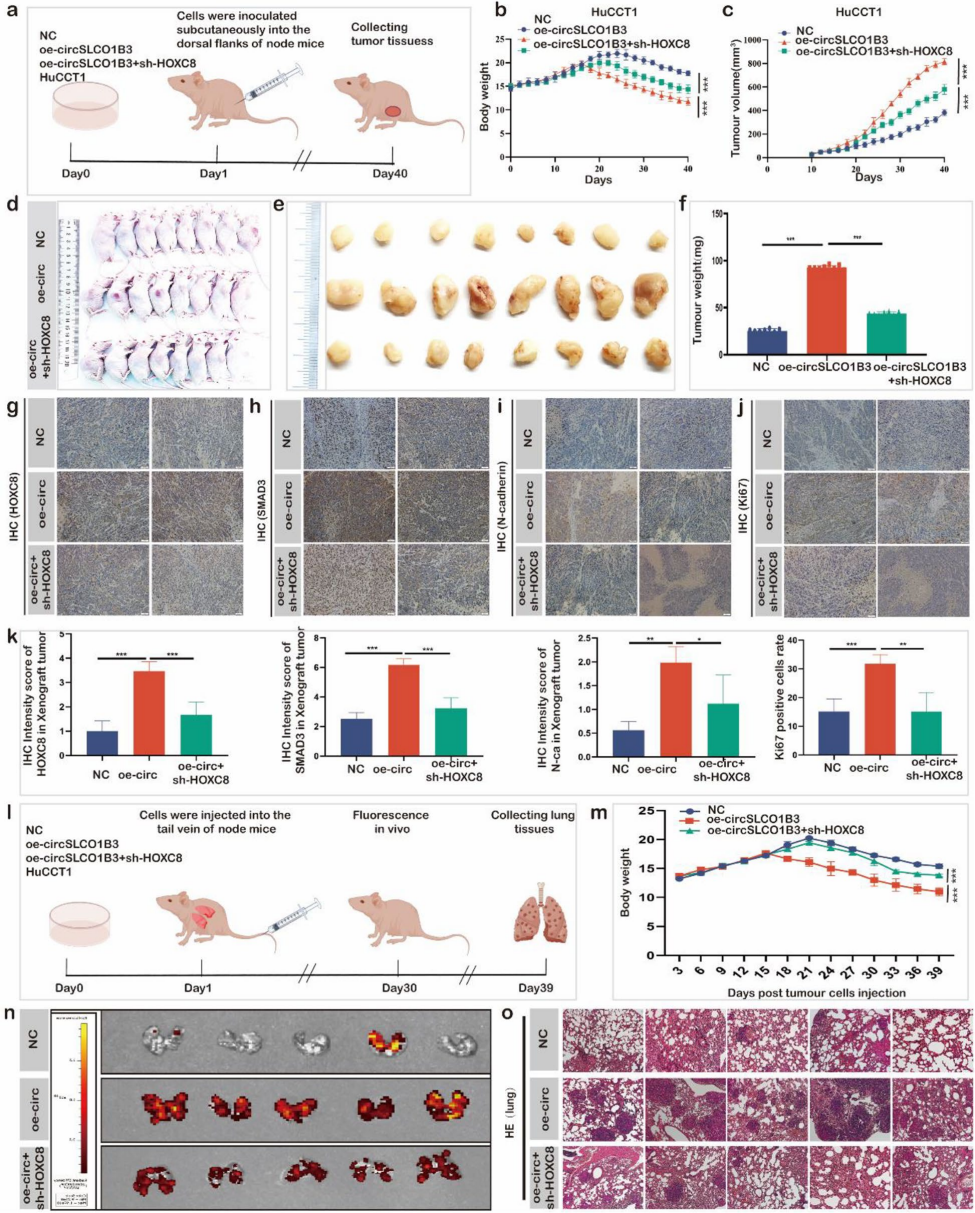
CircSLCO1B3 promoted cholangiocarcinoma progression *in vivo*. **a** The process of constructing subcutaneous xenograft tumor in nude mice**. b**,** c** Mice weight and tumor volumes were monitored every 2 days. Data are mean ± SD and p < 0.05 by one-way ANOVA followed by Tukey’s multiple comparisons test. **d** Stably transfected HuCCT1 cells from different groups were inoculated into BALB/c nude mice to establish subcutaneous xenograft tumors (*n* = 8 mice/group). **e** Images of xenograft tumors of each group (*n* = 8). **f** Tumors weight in three groups. **g**,** h**, **i**,** g**,** k** IHC staining show the relative levels of HOXC8, SMAD3, N-cadherin and ki67 in tumors from different groups. Scale bar, 50 μm. Mean ± SEM. Student's t-test. **l** The process of constructing metastasis xenograft tumour in nude mice. **m** The body weight of the three groups of nude mice. **n** Bioluminescent images of lungs for each experimental group. **o** Pathology of the lung tissue was detected by HE staining. *P < 0.05, 0.001 < ** *P* < 0.01, *** *P* < 0.001

Nude mouse xenografts were generated to reveal how circSLCO1B3 affects ICC growth *in vivo*. HuCCT1 cells were subcutaneously injected into BALB/c nude mice aged 4 weeks with empty vector (NC group) or overexpression plasmid targeting circSLCO1B3 (oe-circSLCO1B3 group) or oe-circSLCO1B3+ HOXC8 shRNA lentiviral plasmid (oe-circSLCO1B3+sh-HOXC8) (Fig. 7a). Mice weight and tumor volumes were assessed every two days. As shown in Fig. 7b, the body weight of nude mice in overexpression circSLCO1B3 group was significantly decreased after 17 days, compared with the control group. Subcutaneously implanted tumors were collected after 40 days. The results showed faster tumor growth and heavier tumors in the overexpression group compared to the vector group, whereas the increased volume and could be counteracted by further treatment with an shRNA-lentiviral plasmid targeting HOXC8 (Fig .7c, d, e, f, Fig.S4a, b). Furthermore, IHC staining were performed on subcutaneous tumor tissues. The results demonstrated that overexpression of circSLCO1B3 increased HOXC8, SMAD3 and N-cadherin ki67 expression. More importantly, the sh-HOXC8 partially counteracted these alterations (Fig .7g, h, i, j, k).

To make further exploration of how circSLCO1B3 influence ICC metastasis, nude mice were injected with stably transfected cells every 3 days for 2 weeks through the tail vein (Fig .7l). Compared with the control group or oe-circSLCO1B3 + shHOXC8 group, there was a significant decrease in body weight experienced by nude mice in the oe-circSLCO1B3 group after 16 days (Fig .7m). After 39 days, lung tissues were harvested. More metastatic nodules on the lungs surface were observed in the oe-circSLCO1B3 group than that in the NC group, and the trend was subsequently reversed by shHOXC8 (Fig. S4c). To observe metastatic nodules deep in lung tissue, we further inspected the fluorescence intensity of the lungs by the small animal *in vivo* fluorescence imaging technology. The results demonstrated the oe-circSLCO1B3 group’s fluorescence intensity was stronger than the NC group’s, which was subsequently decreased by shHOXC8 (Fig .7n). Similarly, according to HE staining results, overexpressed circSLCO1B3 increased the number and volume of pulmonary nodes metastasis in nude mice (Fig .7o). Collectively, these results suggested that circSLCO1B3 could promote ICC growth and metastasis *in vivo* via modulation of HOXC8 expression.

### CircSLCO1B3 promoted ICC immunosuppression via suppressing protein stability of PD-L1 to alleviate CD8^+^ T cell activity

**Fig. 8:**
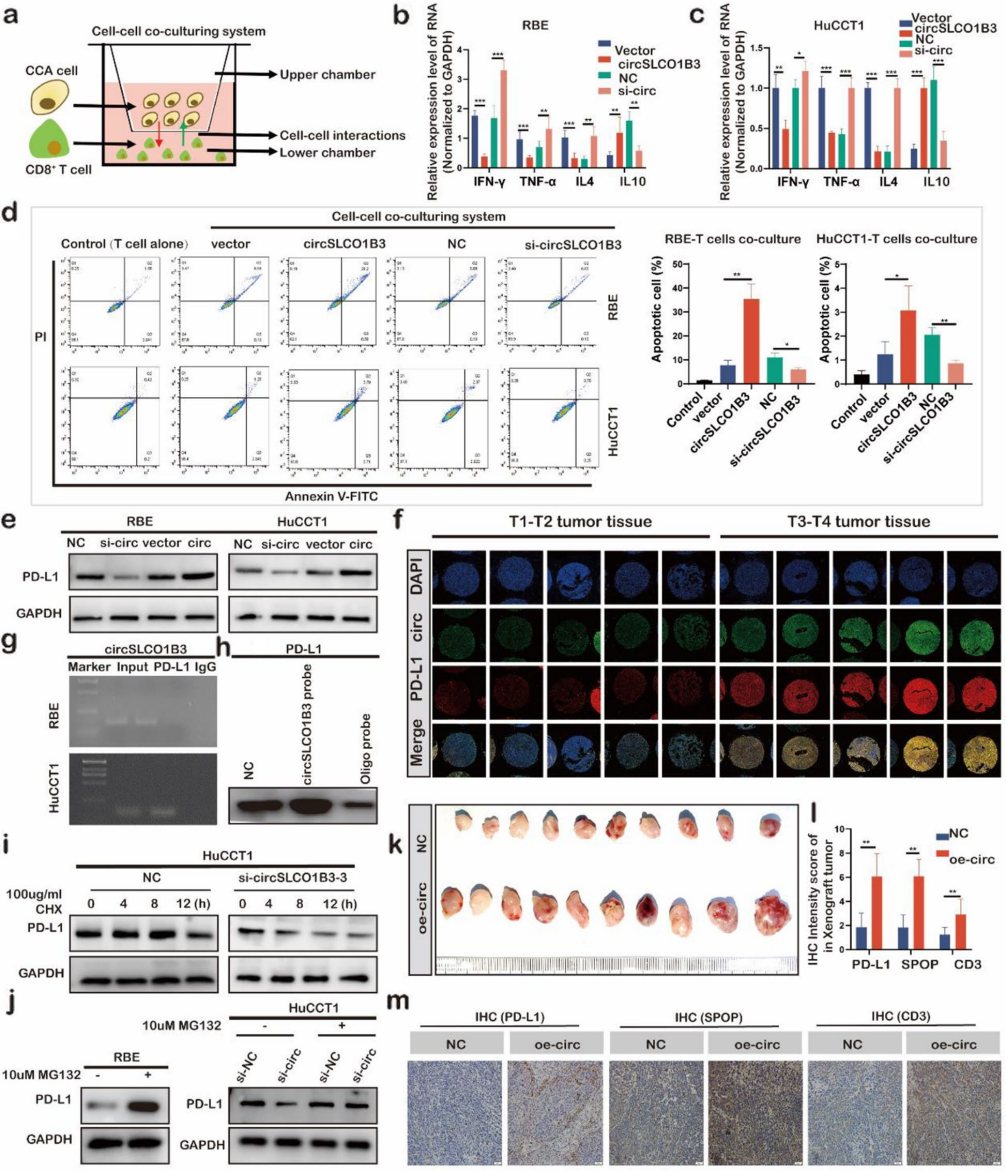
CircSLCO1B3 promotes ICC immunosuppression via suppressing protein stability of PD-L1 to alleviate CD8^+^ T cell activity. **a** The schematic diagram of the trans-well coculturing system. **b**,** c** qPCR was conducted to determine the mRNA levels of IFN-γ, TNF-α, IL-4 and IL-10 in CD8^+^ T cells. **d** FCM was employed to determine the apoptosis ratio of CD8^+^ T cells in the co-culturing system. **e** PD-L1 protein levels were detected by western blotting after depleting or increasing circSLCO1B3 in ICC cells. **f** FISH and IF images of circSLCO1B3 and PD-L1 in ICC tissues (circSLCO1B3-FISH: green spots, PD-L1-IF: red spots, DAPI: blue spots) (magnification, × 200, scale bar, 50 μm). **g** RIP assays were executed to explore the enrichment of circSLCO1B3 by the anti-PD-L1 antibody. **h** CircRNA pull-down was executed in ICC cells to determine the expression levels of PD-L1 by the circSLCO1B3 probe. **i** Western blotting was performed at indicated time points to detect the protein levels of PD-L1 in HuCCT1 cells transfected with circSLCO1B3 siRNA after using cycloheximide. **j** The expression of PD-L1 protein in ICC cells with or without circSLCO1B3 siRNA after treatment with MG-132 by western blot. **k** Images of xenograft tumors of each group for homologous tumor transplantation (*n* = 10). **l**,** m** IHC staining showed the expression levels of PD-L1, SPOP, and CD3 in tumors from different groups. Scale bar, 50 μm. Mean ± SEM. Student's t-test. *P < 0.05, 0.001 < ** *P* < 0.01, *** *P* < 0.001

In addition to the ability of circSLCO1B3 to affect tumor’s proliferation and migration, function enrichment analysis also showed that the differentially expressed genes were enriched in functions related to the immune system. Among all the tumor-infiltrating cell types, CD8^+^ T cells are the most important antitumor members [22, 23]. Therefore, we were interested in whether circSLCO1B3 could affect CD8^+^ T cells. To this end, HuCCT1 cells and RBE cells with deficient or overexpressed of circSLCO1B3 were co-cultured with CD8^+^ T cells purified from healthy human peripheral blood (Fig. 8a). qPCR demonstrated the levels of IFN-γ, TNF-α and IL-4 were increased and IL-10 was inhibited in CD8^+^ T cells when circSLCO1B3 was depleted in ICC cells. Meanwhile, the contrary effect was observed when circSLCO1B3 was overexpressed (Fig. 8b, c). Flow cytometry (FCM) and TUNEL assays demonstrated that CD8^+^ T cells apoptosis was significantly increased after overexpression of circSLCO1B3 and significantly decreased after knockdown of circSLCO1B3 (Fig. 8d, Fig. S5a, b). Moreover, the ELISA assay showed that circSLCO1B3 depletion elevated the secretion of IFN-γ, TNF-α and IL-4 and reduced the secretion of IL-10 in co-cultural supernatants, whereas the contrary effect was observed when circSLCO1B3 was overexpressed (Fig. S5c). The aforementioned findings collectively suggested that circSLCO1B3 promoted the impairment of CD8^+^ T cell function through the promotion of apoptosis, decrease of cytokine secretion, inhibition of IFN-γ, TNF-α, and IL-4 expression, and elevation of IL-10 expression in CD8^+^ T cells.

Blocking PD-1 and PD-L1 immune checkpoints has had remarkable success in antitumor therapy, including ICC [24, 25]. We wonder if circSLCO1B3 can regulate the PD-L1 expression to facilitate dysfunction of CD8^+^ T cells in ICC. To verity the hypothesis, PD-L1 mRNA levels and protein levels were detected. The results of western blotting demonstrated that the knockdown of circSLCO1B3 decreased PD-L1 levels, whereas an overexpression increased them (Fig. 8e). However, qPCR indicated that silencing circSLCO1B3 exerted no effect on the mRNA levels of PD-L1 in HuCCT1 cells and even increased it in RBE cells (Fig. S5d). Moreover, the FISH and IF tests on paraffin-embedded tissue slices which contained 55 ICC tissues indicated a positive association betweenPD-L1 expression and circSLCO1B3 expression in ICC tissues (Fig. 8f, Fig. S5e). In addition, the RIP results suggested that circSLCO1B3 could be enriched significantly more by the PD-L1 antibodies than by IgG (Fig. 8g). The pull-down assays, followed by western blotting, demonstrated that biotin-labeled circSLCO1B3 probe was able to pull down endogenous PD-L1 in RBE and HuCCT1 cells (Fig. 8h).

Considering that circSLCO1B3 alters PD-L1 protein level rather than mRNA level, which indicates it may be achieved by prolongation of protein half-life rather than increased protein synthesis. Thereby, we firstly conducted CHX chase assays and found that PD-L1 protein stability was decreased upon circSLCO1B3 silencing (Fig. 8i, Fig. S5f,). More interestingly, silencing circSLCO1B3 significantly decreased the protein levels of PD-L1, and treating with MG132 could rescued the expression of protein PD-L1 (Fig. 8j). As a result of the assays, circSLCO1B3 was found to bring about possible promotion in the protein expression of PD-L1 by suppressing its proteasome degradation. Thus, immunoprecipitation experiments were subsequently performed and the results revealed that circSLCO1B3 dramatically inhibited ubiquitinated PD-L1 in HuCCT1 cells (Fig. S5g). Next, western blotting was conducted to evaluate the expression of PD-L1 at protein level after depletion of circSLCO1B3 and CSN5, ITCH or SPOP, which were reported as E3 ligase for PD-L1 ubiquitination [26–28]. The results suggested that only the decline of PD-L1 protein level in HuCCT1 via transfecting with circSLCO13 siRNA could be reversed by ablating SPOP (Fig. S5h, i, g). Murine ICC cells were utilized to construct a mouse model for homologous tumor transplantation. Tumor-bearing C57BL/c mice inoculated with over-expressed circSLCO1B3 cells had a faster tumor growth rate (Fig. 8k, Fig. S5k, l) and larger tumor weight (Fig. S5m), which was similar with BALB/c nude tumor-bearing mice model. Immunohistochemical analysis of pathological sections from the tumorigenic area revealed severer local deterioration and greater PD-L1, SPOP and CD3 expression (Fig. 8l, m). Taken together, our results enlightened us to believe that circSLCO1B3 increased the protein expression level of PD-L1 via suppressing the ubiquitin-proteasome pathway to promote immune evasion in ICC.

## Discussion

ICC is a highly malignant cancer with a limited reactivity to traditional chemo-radiotherapy. Despite significant advancements in molecularly targeted treatment over the past five years, patients still have dismal outcomes. There is growing evidence from deep RNA sequencing and bioinformatics that dysregulated circRNAs perform various biological functions in ICC. For instance, increased circNFIB expression influenced MEK1/ERK signaling in ICC cells, which in turn promoted tumor metastasis [29]. CircMBOAT2 accelerated the development of ICC and reprogrammes lipid metabolism by facilitating FASN mRNA cytoplasmic export [9]. In the current study, RNA-seq analysis of the transcriptome revealed that circSLCO1B3 exhibited significant expression in both ICC tissues and cell lines indicating its role as a novel carcinogenic circular RNA. Its expression levels were in positive relation to tumor differentiation, lymph node metastasis, and tumor size. Patients with higher levels of circSLCO1B3 expression had worse prognoses. Furthermore, by conducting gain- and loss-of-function experiments, we verified that circSLCO1B3 facilitated ICC progression not only by modulating ICC proliferation and metastasis, but also by strengthening immune evasion. The findings from all these outcomes indicated that it is justifiable to regard circSLCO1B3 as a potential target and prognostic indicator for ICC patients. Nevertheless, additional research is needed to investigate the specific regulatory mechanisms of circSLCO1B3 in the progression of ICC tumors.

The most extensively researched regulatory mechanism suggests that circRNAs might competitively bind to miRNAs and control target gene expression to carry out their physiological or pathological roles [30–32]. For example, circARFGEF2 sponged miR-1205 and promoted the activation of JAK2, which phosphorylated STAT3 to trigger pancreatic ductal adenocarcinoma lymphangiogenesis and lymph node metastasis [33]. Exosome-delivered CircTGFBR2 functioned as a competitive endogenous RNA by attaching to miR-205-5p to promote ATG5 expression andstimulate autophagy in HCC cells. [34]. In our study, we firstly determined the interaction between circSLCO1B3 and AGO2 by RIP assay. Then, though potential targets prediction and pull-down experiment, miR-502-5p was proved to be the highest enrichment microRNA by circSLCO1B3 probe in ICC cells. Furthermore, dual-luciferase reporter assays provided further insights into the interaction of circSLCO1B3 and miR-502-5p. MiR-502-5p, which is found at Xp11.23, was reported to prevent the advancement of EMT, induce G1 phase arrest, and cause cell death in bladder cancer [35]. In addition, miR-502-5p decreased the aggressiveness of gastric cancer by diminishing PD-L1 expression both at the transcriptional and post-transcriptional levels [36]. In line with these investigations, we demonstrated that miR-502-5p expression was markedly reduced in ICC tissues when compared with adjacent normal tissues and has an attenuating effect in ICC cells’ proliferation and migration. Meanwhile, the qPCR findings indicated a reversed relation between circSLCO1B3 and miR-502-5p in ICC cells. Collectively, these evidences indicated that circSLCO1B3 could directly bind miR-502-5p and impaired its expression to accelerate ICC progression.

Prior researches have demonstrated that TGF- signaling is essential in the development and progression of tumor, invasion, and EMT [37–39]. Among multi-steps of this signal pathways, SMAD proteins act as crucial intracellular effector and was accumulated in the nucleus as transcription factors to promote tumor progression after TGF-β activation. In our study, a series of assays demonstrated that HOXC8 as the target gene of circSLCO1B3 and miR-502-5p could directly bind to the promoter of SMAD3 to induce its expression. SMAD3 belongs to the subclass of facilitated SMADs via forming SMAD 2/3/4 complex in several types of cancer to function in TGF-β signaling [40]. For example, lung cancer metastasis was facilitated by SMYD2-regulated SMAD3 expression in response to TGF-β [41], while colorectal cancer EMT was inhibited by miR-140 by directly downregulating SMAD3 and deactivating the TGF-β signaling pathway [42]. In this study, dual luciferase assay, ChIP assay and qPCR confirmed the interaction between HOXC8 and SMAD3. Base on the above, there’s reason to believe that circSLCO1B3 promotes proliferation, invasion and EMT via miR-502-5p/HOXC8/SMAD3 axis in ICC cells.

There is growing data to reinforce our understanding of the ICC tumor microenvironment, which has a significant number of immune cells to facilitate tumor immune evasion [43]. These immune cells mainly include tumor-associated macrophages, myeloid-derived suppressor cells (MDSCs), tumor-associated neutrophiles and T cells [2, 44]. For example, ALKBH5 prevented the expansion and cytotoxicity of T cells by maintaining PD-L1 expression in ICC cells to promote immune evasion [45]. Human CXCL8 translation was specifically controlled by METTL1-mediated m7G tRNA alteration, allowing MDSC to accumulate in the tumor immunological environment to accelerate ICC progression *in vivo* [46]. However, the complete understanding of the clinical significance and regulatory systems of circRNAs in cancer patients undergoing immune checkpoint blockades remains unclear. In this study, we identified circSLCO1B3 could upregulate the expression of PD-L1 to inactive the CD8^+^ T cells. In this regard, circSLCO1B3 could strengthen ICC cells evasion from immune survivance by modulating PD-L1. Then, the molecular mechanism how circSLCO1B3 regulated PD-L1 was further revealed. In the previous literature, circRNAs usually were reported to regulate transcription of PD-L1 by binding microRNA. Such as, circTMTC3 and circFAM117B reduced T cell activity by inhibiting miR-142-5p to increase PD-L1 expression in melanoma [47]. CircCPA4 facilitated immune evasion by acting as an RNA sponge for let-7 miRNA to downregulate PD-L1 in non-small cell lung cancer [48]. However, in our study, qPCR and western blotting verified circSLCO1B3 was only regulates the protein level of PD-L1 rather than mRNA level. Therefore, we deduced circSLCO1B3 might regulate immune evasion by interacting with RNA binding proteins. The results of pull-down assay and RIP assay affirmed this conjecture. Moreover, circSLCO1B3 was confirmed to increase PD-L1 expression through inhibiting the ubiquitination degradation. Although PD-L1 has been reported to be ubiquitinated by CSN5, SPOP, ITCH and so on [49–51], the regulatory effect of circSLCO1B3 on PD-L1 expression was blocked only by SPOP silencing in this study. In this regard, our results demonstrated that circSLCO1B3 could be a potential therapeutic target to facilitate immune evasion in ICC by regulating the protein level PD-L1. However, PD-L1/PD-1 pathway is only one of the ways to regulate tumor immune escape [52]. In the future, we will explore more about circSLCO1B3 regulating immune escape in cholangiocarcinoma.

Given the important role of circSLCO1B3 in the progression of cholangiocarcinoma, we continued to investigate the reasons for its abnormal expression. The m6A modification, which is one of the frequently occurring internal RNA modifications, is linked to circRNAs metabolism in various aspects [53]. Previous research verified that YTHDF2 has the ability to control the expression of circPOLR2A in a manner involving m6A modification in clear renal cell carcinoma [54]. METTL3-mediated m6A modification stabilized circQSOX1 in colorectal cancer [55]. In this study, we found that METTL3-mediated m6A modification on circSLCO1B3 could be read by YTHDC1 in ICC cells. A report indicated that METTL3 and YTHDC1 direct the back-splicing reaction of circZNF609 in a m6A-midofied manner [56]. However, YTHDC1 and METTL3 in our study strengthened the stability of circSLCO1B3 rather than promoted back-splicing in ICC. Furthermore, we found that METTL3 high expressed in ICC cells and could promote proliferation, migration, and invasion. This was consistent with previous research [14]. Therefore, METTL3 and YTHDC1 mediated m6A modification made circSLCO1B3 more stable in ICC, which revealed that targeting m6A may be an effective strategy to inhibit circSLCO1B3 expression in ICC.

## Conclusion

**Fig. 9:**
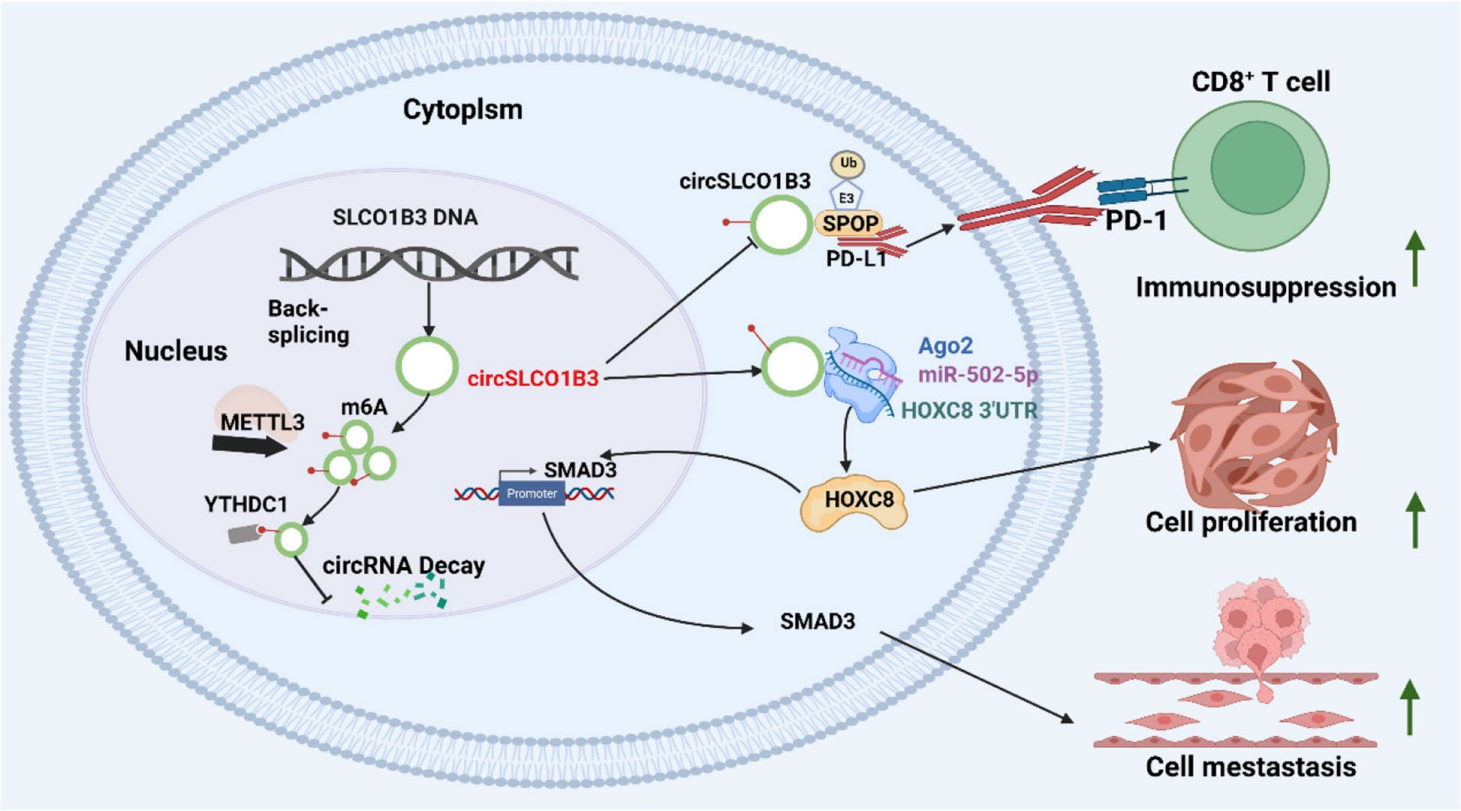
A schematic model of circSLCO1B3 function in tumor proliferation, metastasis and immunosuppression.

In conclusion, we found that circSLCO1B3 was correlated with poor prognosis in ICC and could promote the proliferation, migration and invasion via the miR-502-5p/HOXC8/SMAD3 axis, more than that, it facilitated immune evasion via antagonizing PD-L1 degradation in ICC. We also found METTL3-mediated m6A modification on circSLCO1B3 could be read by YTHDC1 in ICC cells (Fig. 9). Therefore, circSLCO1B3 plays a vital role in cholangiocarcinoma progression and has significant potential as a molecular target for cholangiocarcinoma ICC therapy.

### Supplementary Information

**Supplementary Material 1.**

## Data Availability

The datasets used and/or analyzed during the current study are available from the corresponding author on reasonable request.

## Abbreviations

ICC: Intrahepatic cholangiocarcinoma
circRNAs: Circular RNAs
qPCR: Quantitative real-time PCR
RIP: RNA immunoprecipitation
ChIP: Chromatin immunoprecipitation
gDNA: Genomic DNA
cDNA: Complementary DNA
siRNAs: Small-interfering RNAs
miRNAs: MicroRNAs
mRNA: Messenger RNA
ECL: Enhanced chemiluminescence
FISH: Fluorescence in situ hybridization
IF: Immunofluorescence
TMA: Tissue microarray
siRNAs: Small interfering RNAs
RIPA: Radioimmunoprecipitation assay buffer
BCA: Bicinchoninic Acid Assay
SDS-PAGE: Sodium Dodecyl Sulfate PolyAcrylamide Gel Electrophoresis
PVDF: Polyvinylidene fluoride
RIP: RNA immunoprecipitation assay
MeRIP: Methylated RNA immunoprecipitation
AGO2: Argonaute 2
FCM: Flow cytometry
ELISA: Enzyme-linked immunosorbent assay
SD: Standard Deviation
ROC: Receiver operating curve
GO: Gene Ontology
KEGG: Kyoto Encyclopedia of Genes and Genomes
EMT: Epithelial-mesenchymal transition
TNM: Tumor Node Metastasis
m6A: N6-methyladenosine
DAPI: 4',6-diamidino-2-phenylindole
CCK-8: Cell counting Kit-8
CHX: Cycloheximide
IgG: Immunoglobulin G
