# Supplementary material for: N6-methyladenosine-modified circSLCO1B3 promotes intrahepatic cholangiocarcinoma progression via regulating HOXC8 and PD-L1
Source: J Exp Clin Cancer Res. 2024 Apr 20;43:119. doi: 10.1186/s13046-024-03006-x (PMC11031933; doi:10.1186/s13046-024-03006-x)
Supplement: Supplementary file 1 — Supplementary Material 1. [file 13046_2024_3006_MOESM1_ESM.pdf]

Supplementary Figs

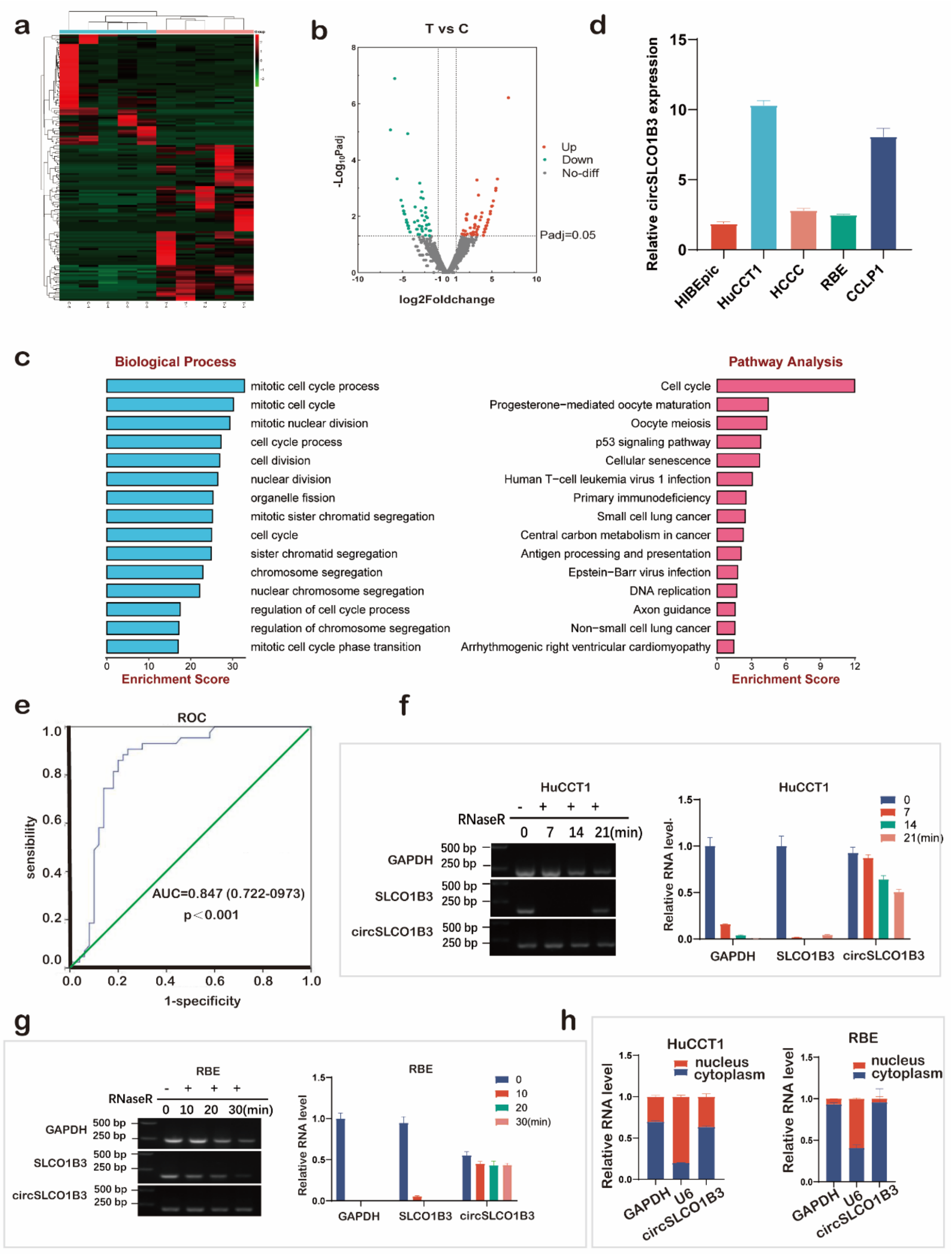

Fig. S1 CircSLCO1B3 validated and characterized in ICC cells. **a** Cluster heat map of differentially expressed circRNAs transcripts in 5 pairs of cancer and adjacent tissues of patients with ICC via RNA-seq analysis. **b** Volcano plot illustrated the expression profile between five pairs of tumor tissues and adjacent non-neoplastic tissues (T, tumor,

N, normal). **c** GO function analysis and KEGG pathway analysis of differently expressed circRNAs. **d** The expression of circSLCO1B3 in HIBEpic and ICC cells. **e** ROC curve was plotted according to FISH scores of paraffin-embedded tissue sections containing 93 pairs of CCA and matched adjacent normal tissues. **f, g** The stability of circSLCO1B3 were detected by qPCR and agarose gel electrophoresis using RNase R. **h** QPCR analysis was employed for nuclear and cytoplasmic circSLCO1B3.

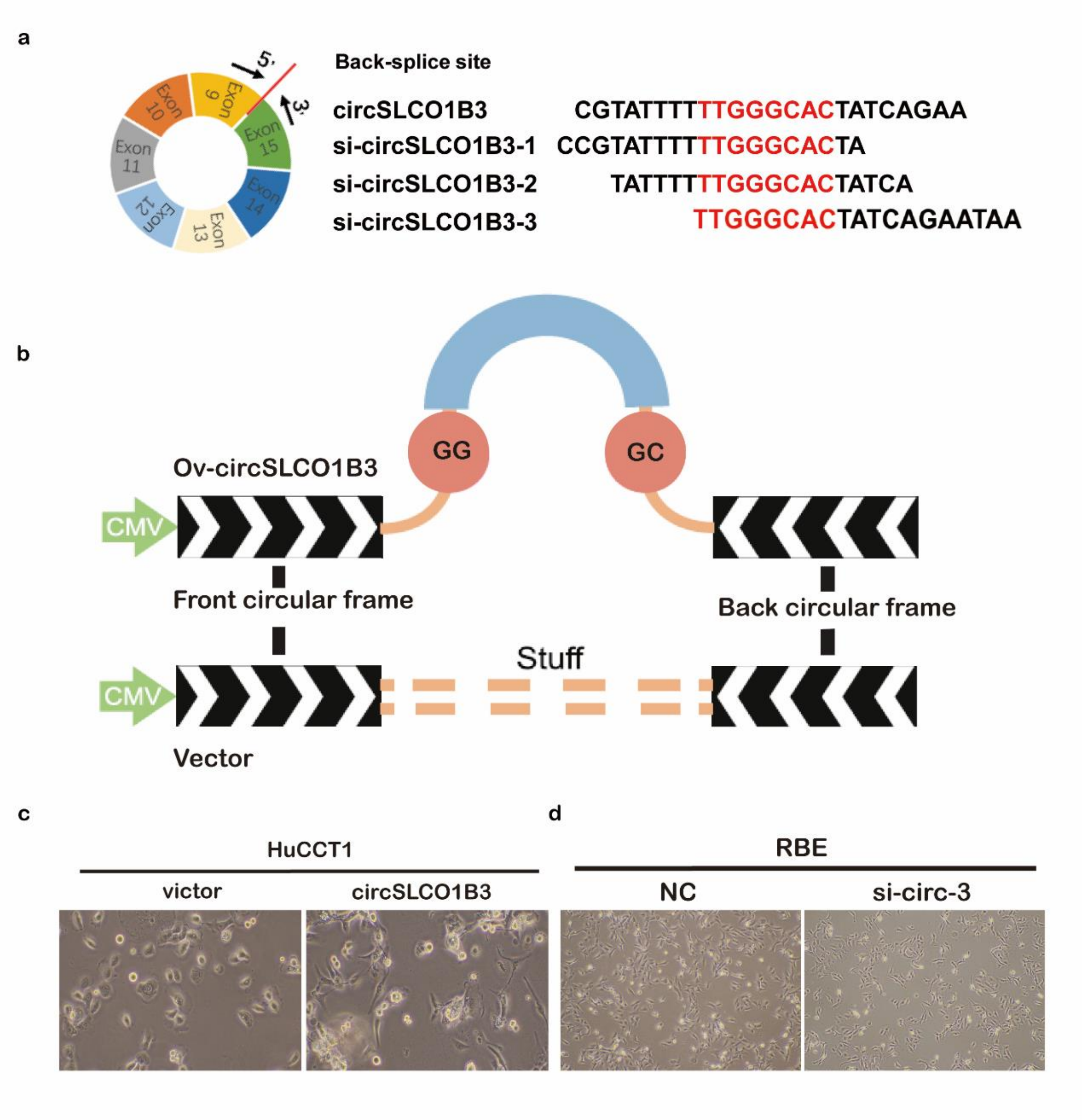

Fig. S2 CircSLCO1B3 functions to induce ICC cells morphological change. **a, b** The schematic illustration of small interfering RNAs (siRNAs) and circSLCO1B3 expression vector specifically targeting the backsplice junction sequences. **c, d** The cell morphological change of ICC cells with depletion or overexpression of circSLCO1B3.

a

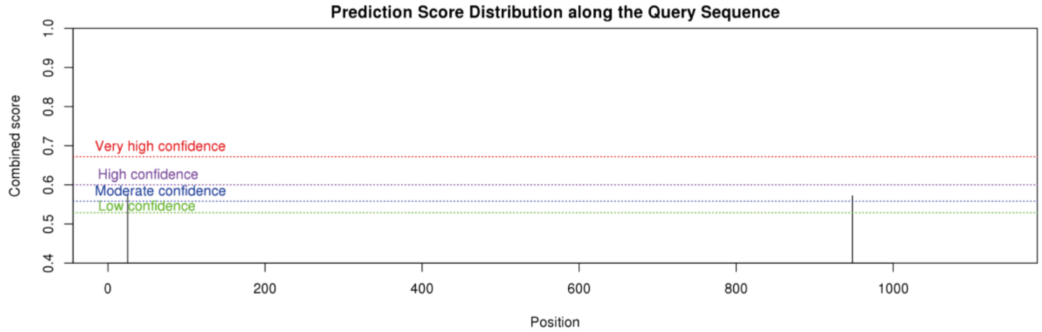

#	Position	Sequence context	Structural context	Local structure visualization	Score(binary)	Score(knn)	Score(spectrum)	Score(combined)	Decision
1	25	ACUAU CAGAA UAACU<br>CCUAA GGACU CUCGU<br>UGGGU UGGAG CUUGG	N/A	N/A	0.721	0.337	0.406	0.575	m^6A site (**Moderate** confidence)
2	948	AGGUA CCACA UUUAU<br>CUUGU UGACU GUGAA<br>GAUUG UUCAA CCUGA	N/A	N/A	0.567	0.434	0.597	0.572	m^6A site (**Moderate** confidence)

b c

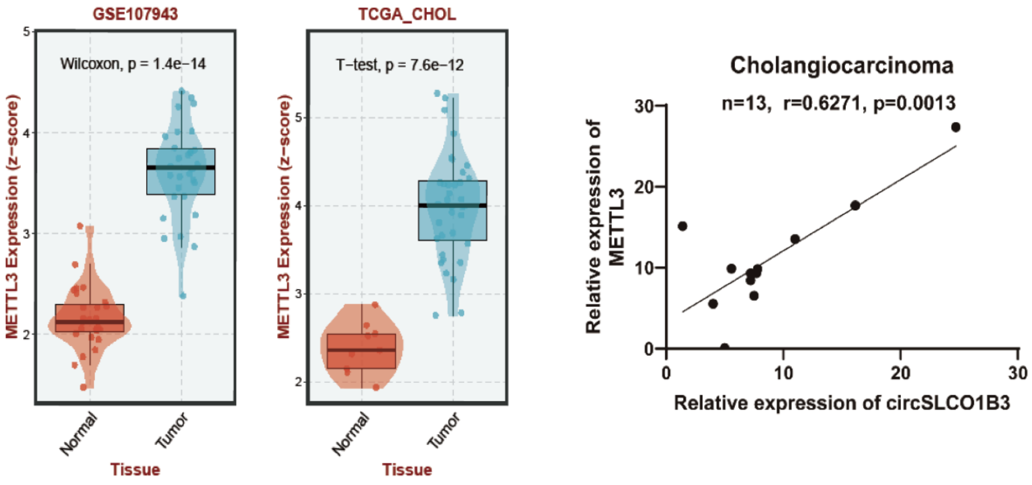

Fig. S3 METTL3-mediated m6A modification exists on circSLCO1B3. **a** The online website SPAMP was used to predict m6A site in circSLCO1B3. **b** Methyltransferase METTL3 was significantly up-regulated GEO and GEPIA2 dataset. **c** The results of qRT-PCR in 13 pairs of CCA tissues and matched adjacent normal tissues showed the relation METTL3 and circSLCO1B3 expression.

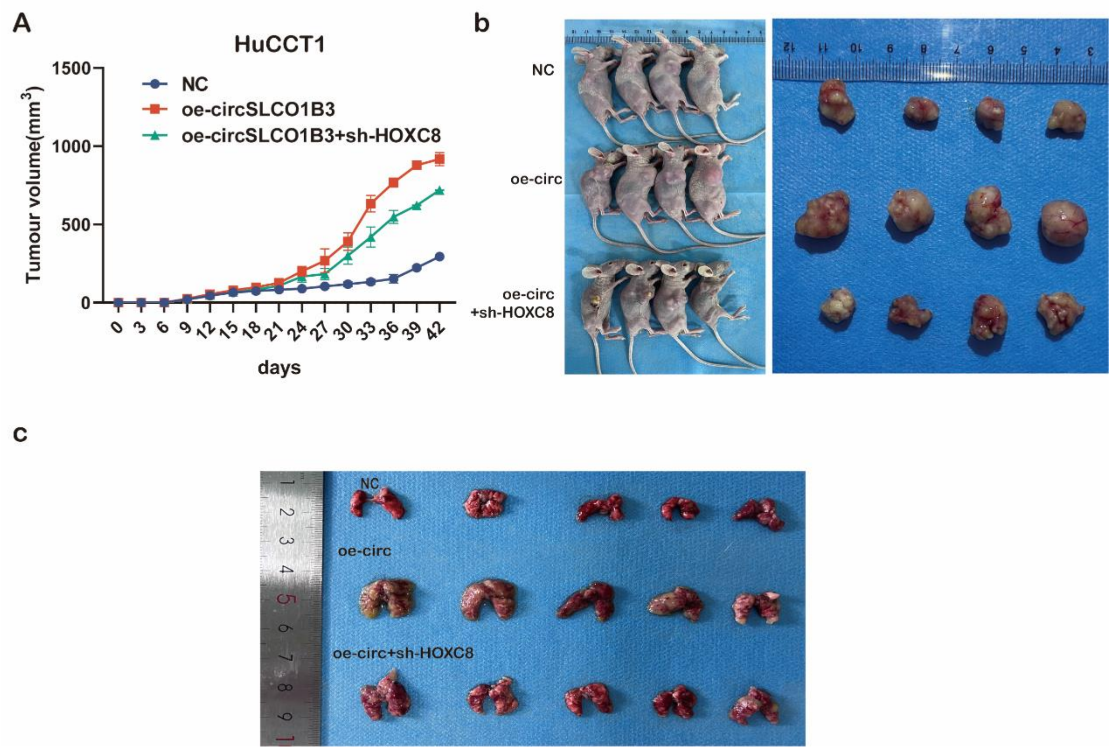

Fig. S4 CircSLCO1B3 promoted cholangiocarcinoma progression in vivo. **a** Tumor volume was monitored every 3 days. **b** Images of xenograft tumors of each group (n = 4).

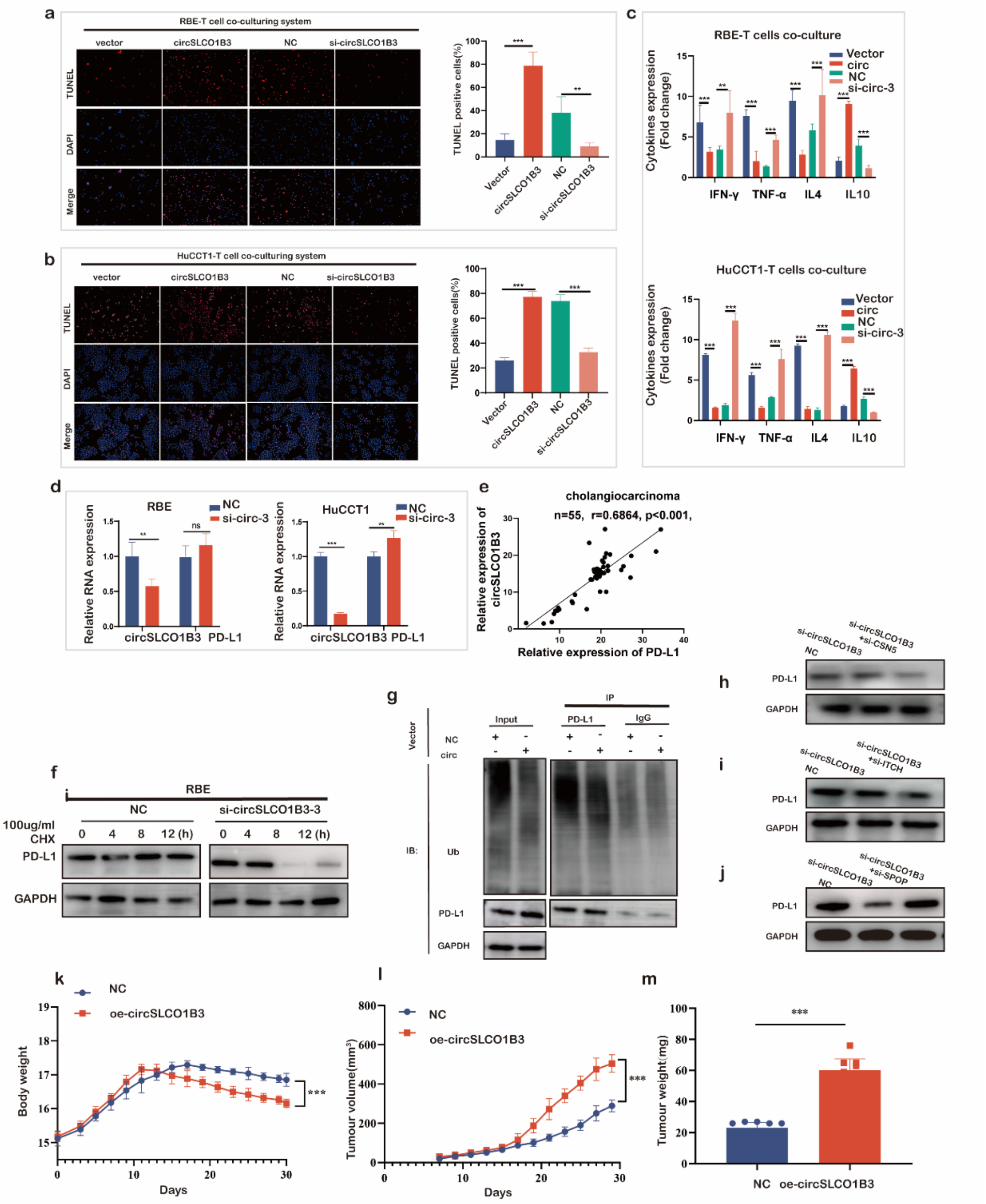

Fig. S5 CircSLCO1B3 promotes ICC immunosuppression via suppressing protein stability of PD-L1 to alleviate CD8+ T cell activity. **a, b** TUNEL staining of CD8^{+} T cells after coculturing with circSLCO1B3 vectors or circSLCO1B3-silenced ICC cells. **c** The expression levels of IFN-γ, TNF-α, IL-4 and IL-10 in the supernatants of the co-culturing system were determined by using ELISA assay. **d** PD-L1 mRNA levels were detected by qPCR after depleting or increasing circSLCO1B3 in ICC cells. **e** The correlation of HOXC8 and circSLCO1B3 in FISH and IF scores. **f** Western blotting

was performed at indicated time points to detect the protein levels of PD-L1 in RBE cells transfected with circSLCO1B3 siRNA after using cycloheximide. **g** The ubiquitination of PD-L1 in ICC cells with circSLCO1B3 overexpression. **h, i, j** Western blot analysis of PD-L1 in ICC cells transfected with or without si-circRNASLCO1B3 or si-CSN5, si-ITCH and si-SPOP. **k, l** Mice weight and tumor volumes were monitored every 2 days. **m** Tumors weight in three groups. *$P < 0.05$, $0.001 <$ ** $P < 0.01$, *** $P < 0.001$.

Table S1 Personal characteristics of the patients who donated tissues for sequencing

Sex	Age	Tumour size (cm)	Tumour type	Tumour differentiation	TNM stage	metastasis	Previous history of treatment
M	63	2.7	Adenocarcinoma	Moderate differentiation	T1N0M0	No lymph node metastasis	None
F	51	3.5	Adenocarcinoma	Moderate differentiation	T2N1M0	Local lymph node metastasis	None
M	59	1.6	Adenocarcinoma	Moderate differentiation	T1N0M0	No lymph node metastasis	None
M	60	2.1	Adenocarcinoma	Moderate differentiation	T2N1M0	Local lymph node metastasis	None
F	57	1.8	Adenocarcinoma	Moderate differentiation	T3N2M0	Local lymph node metastasis	None

Table S2 Sequences of the qPCR primers used in this study

Gene name	Forward primer (5'-3')	Reverse primer (5'-3')
circSLCO1B3 Convergent	CACACTTGGGTGAATGCCCA	ATGTGGTACCTCCTGTTGCAG
circSLCO1B3 Divergent	TGCAATGGGTTTCCAGTCAAT	CCAACCCAACGAGAGTCCTTA
SLCO1B3	CTTGCAATGGGTTTCCAGTCA	GCCCAAGTAGACCCTTCCAA
HOXC8	ACCGGCCTATTACGACTGC	TGCTGGTAGCCTGAGTTGGA
NFYA	CAGTGGAGGCCAGCTAATCAC	CCAGGTGGGACCAACTGTATT
ATXN7	GAACTCCCTGGCGCCTCC	GATTCCACGACTGTCCCAGCAT
DAZAP2	ACCATGTCAGCCGCAATCC	TCTGGCACCTGCATCATACC
B3GALT5	TGGGTCCATCGCTTTTGTCC	GCTGCCTGATGGGAAATGCG
RAB1B	GGACTTCAAGATCCGAACCAT	ATACACCACGATGATGCCA
RPL36A-HNRNPH2	TGCTAAGGCTTGAGTGCGTT	TGCAATTTTGGTGTAGCTTTCTC
OFD4	GCTTATCCTATACTTCAAATGCG	GCCAGGAGTTCAGAAAAGATTACAC
IGF1R	GGCACAATTACTGCTCCAAAGAC	CAAGGCCCTTTCTCCCCAC
TNRC6C	CCGTTGCTTGGTCCAGTTTC	TGCTGCTGTGCCATTATCCA
CTSB	TGTAATGGTGGCTATCCTGCT	AGGCTCACAGATCTTGCTACA
CALM1	CGGTTGGAGATGTTGAGGCTGA	GACCCAGTGACCTCATGACAG
N-cadherin	AGCCAACCTTAACTGAGGAGT	GGCAAGTTGATTGGAGGGATG
E-cadherin	CGAGAGCTACACGTTCACGG	GGGTGTCGAGGGAAAAATAGG
Vimentin	TCCGCACATTCGAGCAAAGA	TGATTCAAGTCTCAGCGGGC
SNAIL	TCGGAAGCCTAACTACAGCGA	AGATGAGCATTGGCAGCGAG
ZEB1	GCACCTGAAGAGGACCAGAG	GTGTAACTGCACAGGGAGCA
SMAD3	CTAGAGTCAGGAGCAGGGACT	ACTCTAGGAAGCCAAGGGGA
METTL3	GTGATCGTAGCTGAGGTTCGT	GGGTTGCACATTGTGTGGTC
YTHDC1	ATCTTCCGTTCGTGCTGTCC	GGACCATACACCCTTCGCTT
pre-SLCO1B3	GGATCTAAACTACACAGACCGAAG	ggctcagagctgtttaacacttac
SMAD4	CCCATCCCGGACATTACTGG	TGTGCAACCTTGCTCTCTCAA
PD-L1	TGGCATTTGCTGAACGCATTT	TGCAGCCAGGTCTAATTGTTTT
GAPDH	AACGGATTTGGTCGTATTGG	TTGATTTTGGAGGGATCTCG
U6	GGAACGATACAGAGAAGATTA	TGGAACGCTTCACGAATTTGCG

Table S3 Sequences of the siRNA used in this study

Gene name	Sequences（5'to 3'）
si-circSLCO1B3-1	CCGTATTTTTTGGGCACTA
si-circSLCO1B3-2	TATTTTTTGGGCACTATCA
si-circSLCO1B3-3	TTGGGCACTATCAGAATAA
si-HOXC8	GCAATATCCCGACTGTAAATC
si-METTL16	CAGTTCCCTTGAGACTCAACTATAT
si-METTL3	GGAAGAGAAGACCTTACAA
si-METTL4	GGAGTTCACTACTTCTGTT
si-YTHDC1	GGAGAAAGATGGAGAACTT
si-ALKBH5	TCAACAGCGCCGTCATCAA
si-FTO	GGAGCTCCATAAAGAGGTT
si-YTHDF2	CCTACCAGATGCAATGTTT
si-YTHDF3	GAGCCATACTTAAGTAGCCAGACAA

Table S4 Primer pairs of the SMAD3 promoters used in this study

Primer name	Sequences（5’to 3’）	Amplified product （bp）
h-SMAD3 -promoter-F1	GCCTCATTACCACGTCACAC	179
h-SMAD3 -promoter-R1	GATGCCCCTCATTCAGGCAC	
h-SMAD3 -promoter-F2	TGTTGAATGGCTACAGGCCC	107
h-SMAD3 -promoter-R2	GTGCCCAAGCAAGAACTGTC	
h-SMAD3 -promoter-F3	GCATGGACCTCTGTCCTCATT	81
h-SMAD3 -promoter-R3	GGGCTACAAGAGTGGTCCTG	
h-SMAD3 -promoter-F4	TATGAGCTTGTGCTTGCTGGA	122
h-SMAD3 -promoter-R4	CTCTTGCCCACATGTTGCTC	
